# Deletion of *Fmr1* in parvalbumin-expressing neurons results in dysregulated translation and selective behavioral deficits associated with fragile X syndrome

**DOI:** 10.1186/s13229-022-00509-2

**Published:** 2022-06-29

**Authors:** Magdalena Kalinowska, Mathijs B. van der Lei, Michael Kitiashvili, Maggie Mamcarz, Mauricio M. Oliveira, Francesco Longo, Eric Klann

**Affiliations:** 1 Center for Neural Science, New York University, New York, NY USA; 2 Department of Medical Genetics, University of Antwerp, Antwerp, Belgium; 3 Institute for Neuroscience and Physiology, University of Gothenburg, Gothenburg, Sweden; 4 Wallenberg Centre for Molecular and Translational Medicine, University of Gothenburg, Gothenburg, Sweden; 5 NYU Neuroscience Institute, New York University Langone Medical Center, New York, NY USA

**Keywords:** FMRP, Fragile X syndrome, Inhibitory neurons, Behavior, Autism, Protein synthesis

## Abstract

**Background:**

Fragile X syndrome (FXS), the most common genetic cause of autism spectrum disorder and intellectual disability, is caused by the lack of fragile X mental retardation protein (FMRP) expression. FMRP is an mRNA binding protein with functions in mRNA transport, localization, and translational control. In *Fmr1* knockout mice*,* dysregulated translation has been linked to pathophysiology, including abnormal synaptic function and dendritic morphology, and autistic-like behavioral phenotypes. The role of FMRP in morphology and function of excitatory neurons has been well studied in mice lacking *Fmr1*, but the impact of *Fmr1* deletion on inhibitory neurons remains less characterized. Moreover, the contribution of FMRP in different cell types to FXS pathophysiology is not well defined. We sought to characterize whether FMRP loss in parvalbumin or somatostatin-expressing neurons results in FXS-like deficits in mice.

**Methods:**

We used Cre-lox recombinase technology to generate two lines of conditional knockout mice lacking FMRP in either parvalbumin or somatostatin-expressing cells and carried out a battery of behavioral tests to assess motor function, anxiety, repetitive, stereotypic, social behaviors, and learning and memory. In addition, we used fluorescent non-canonical amino acid tagging along with immunostaining to determine whether de novo protein synthesis is dysregulated in parvalbumin or somatostatin-expressing neurons.

**Results:**

De novo protein synthesis was elevated in hippocampal parvalbumin and somatostatin-expressing inhibitory neurons in *Fmr1* knockout mice. Cell type-specific deletion of *Fmr1* in parvalbumin-expressing neurons resulted in anxiety-like behavior, impaired social behavior, and dysregulated de novo protein synthesis. In contrast, deletion of *Fmr1* in somatostatin-expressing neurons did not result in behavioral abnormalities and did not significantly impact de novo protein synthesis. This is the first report of how loss of FMRP in two specific subtypes of inhibitory neurons is associated with distinct FXS-like abnormalities.

**Limitations:**

The mouse models we generated are limited by whole body knockout of FMRP in parvalbumin or somatostatin-expressing cells and further studies are needed to establish a causal relationship between cellular deficits and FXS-like behaviors.

**Conclusions:**

Our findings indicate a cell type-specific role for FMRP in parvalbumin-expressing neurons in regulating distinct behavioral features associated with FXS.

**Supplementary Information:**

The online version contains supplementary material available at 10.1186/s13229-022-00509-2.

## Background

Fragile X syndrome (FXS) is a heritable developmental disorder resulting from abnormal trinucleotide CGG repeat expansion and transcriptional silencing of the X-linked *FMR1* gene that encodes fragile X messenger ribonucleoprotein 1 (FMRP) [1, 2]. FXS is the most prevalent known genetic cause of autism spectrum disorder (ASD) and intellectual disability (ID), with approximately 2–5% of all ASD cases linked to FXS, and 85% of males and 25% of females with FXS exhibiting ID [3, 4]. Clinical presentation of FXS is complex, ranging from normal functioning (mainly in mosaic females) to severe ID and ASD, and is associated with additional psychiatric conditions, including anxiety, social avoidance, self-injurious behavior and hyperactivity/impulsivity, which are generally more severe in males [1, 2, 5]. Overall, more than half of males and ~ 20% of females with FXS meet the *Diagnostic and Statistical Manual of Mental Disorders* (5th edition, DSM-V) criteria for ASD diagnosis based on impairments in communication and social interaction, and presentation of restrictive, repetitive behaviors [3, 4]. Even though much research has been devoted to the biology and cellular function of FMRP, the mechanism of how reduction or loss of FMRP leads to ASD symptoms has not been clearly elucidated.

FMRP is an mRNA binding protein that can regulate mRNA translation, stability and transport, and was shown to bind > 800 mRNAs encoding cellular and synaptic proteins in the mouse brain, many of which have been identified as ASD susceptibility genes [6–10]. FMRP typically functions as a repressor of translation in cells and its loss results in global elevation of protein synthesis in neurons and leads to aberrant synaptic structure, function, and plasticity [11, 12]. Many of the behavioral features associated with FXS, such as impairments in social interaction and repetitive behaviors, can be modeled in *Fmr1* knockout mice (*Fmr1*^−/y^ mouse) [13]. Furthermore, the pharmacological and genetic manipulation of several proteins involved in translational regulation was shown to rescue molecular, synaptic, and behavioral deficits observed in *Fmr1*^−/y^ mouse, including restoration of elevated protein synthesis [12, 14–16].

The link between aberrant protein synthesis and autistic-like behavioral deficits has been demonstrated in mice by studies utilizing genetic manipulation of proteins involved in cap-dependent translation, and recently it has been demonstrated that cell type-specific deletion of the translational repressor eukaryotic translation initiation factor 4E-binding protein 2 (4E-BP2) specifically in γ-aminobutyric acid (GABA)ergic inhibitory neurons results in autistic-like behavioral alterations in mice [17–19]. There is a large diversity of GABAergic neuron subtypes in the neocortex that vary in morphology, electrophysiological properties, connectivity patterns and expression of specific molecular markers. Ca^2+^-binding protein parvalbumin (PV) and the neuropeptide somatostatin (SOM)-expressing inhibitory neurons are the major inhibitory neuron subtypes in CNS, accounting for 40% and 30% of all GABAergic neurons, respectively [20]. PV-positive inhibitory neurons are fast-spiking basket and chandelier cells, targeting soma and axon initial segment of neurons where they provide strong inhibition of synaptic firing, whereas SOM-expressing Martinotti cells target distal dendrites to gate inputs to pyramidal neurons [21]. Recent studies indicate that these major inhibitory neuron subtypes play very distinct roles in neural circuits and perform different functions in behaving animals. For example, in medial prefrontal cortex (mPFC), SOM, but not PV, neuron activity is critical for discrimination of affective states during social interaction, whereas PV neuron activity is necessary for social investigation behavior [22]. In the amygdala, during threat conditioning, PV and SOM neurons are activated during distinct behavioral phases to exert bidirectional control on acquisition of fear [23]. Cell type-specific function of FMRP and its role in the performance of behavioral tasks, as well as its contribution to FXS-associated behavioral deficits has not been examined in detail.

Here, we used Cre-lox recombinase technology to assess whether deletion of *Fmr1* in either PV or SOM-expressing neurons leads to behavioral deficits in mice. We found that cell type-specific deletion of *Fmr1* in PV, but not SOM-expressing neurons, results in behavioral alterations, including anxiety-like behavior and deficits in social interaction. Furthermore, FMRP loss from PV-positive neurons was associated with brain region-specific dysregulation of de novo protein synthesis, whereas its absence from SOM-positive neurons did not impact global protein synthesis in mouse cortex or hippocampus. Our findings uncover a distinct cell type-specific role for FMRP in two major inhibitory neuron populations in mediating specific cellular and behavioral deficits associated with FXS.

## Materials and methods

### Mice

All procedures involving mice were approved by New York University (NYU) Animal Care and Use Committee and followed the National Institutes of Health Guidelines for use of animals in research. Adult male mice (2–6 months of age) were used for behavioral experiments, fluorescent non-canonical amino acid tagging (FUNCAT) and immunocytochemistry in brain slices. All mice were housed in the NYU animal facilities and kept on a regular 12 h light/dark cycle (lights on at 7:00 am) with food and water provided ad libitum. Adult mice were handled once per day for 2 days before starting behavioral testing.

### Generation of *Fmr1* conditional knockout mice

To generate mice with cell-type specific deletion of *Fmr1*, *Fmr1*^*flx/flx*^ mice [24] were crossed with mice expressing Cre recombinase under cell type-specific promoters. Pv-Cre (129P2-Pvalb^tm1(cre)Arbr^/J, Jackson lab stock#008069) is a knock-in mouse line that expresses Cre recombinase in parvalbumin-expressing neurons without disrupting endogenous *Pvalb* expression. Som-Ires-Cre (Sst-IRES-Cre, Jackson lab stock#013044) is a knock-in mouse line that expresses Cre recombinase in somatostatin-expressing neurons without disrupting endogenous *Sst* expression. The Cre-positive flx/ + heterozygous progeny was subsequently crossed to each other to generate the following conditional KO (cKO) strains: *Fmr1*^flx/flx^:Pv-Cre (*Fmr1*^−/y^-PV) and *Fmr1*^flx/flx^:Som-Ires-Cre (*Fmr1*^−/y^-SOM). To account for any effects generated by presence of Cre recombinase, cKO mice were compared to littermate Fmr1^+/+^:Pv-Cre and Fmr1^+/+^:Som-Ires-Cre (both referred here as “WT-PV” and “WT-SOM”, respectively).

### Behavioral test battery

A battery of behavioral tests was used to evaluate ASD-like behaviors, learning and memory function as described previously [25, 26]. For all behavioral experiments, mice were habituated for 30 min to experimental rooms prior to behavioral testing and all behavioral apparatuses were cleaned with 30% ethanol before each trial. The experimenter was blind to mouse genotype during performing and scoring of all behavioral tasks. Behavioral testing was performed starting with the least stressful task first and ending with the most aversive test in the following order: rotarod, elevated plus maze, marble burying, self-grooming, novel object recognition, object location memory, three chamber social interaction (3CSI) test, Morris water maze, water-based Y maze, and lastly, associative threat conditioning.

#### Rotarod

Motor coordination and learning were assessed using an accelerating rotarod (UGO Basile, Varese, Italy). Mice were placed on a rotating drum which gradually accelerated from 4 to 42 r.p.m. over a 300 s period and a latency to fall off the rotarod was recorded (max 300 s). Four trials with an inter trial interval (ITI) of 15 min were performed on two consecutive days.

#### Elevated plus maze

Anxiety-like behavior was assessed using a plus-shaped maze apparatus with two open arms (30 cm × 5 cm) and two contralateral enclosed arms (30 × 5 × 15 cm). Mice were placed in a center of the maze and their activity was recorded for 5 min with video tracking system (Ethovision). Time spent in open and closed arms and number of entries made into each arm were calculated using tracking software.

#### Marble burying

Mice were placed individually in clear plexiglass cages containing 3 cm of fresh bedding with 20 clean marbles prearranged in a 4 by 5 grid. Mice were allowed 30 min to bury marbles and their activity was recorded using video tracking system (Ethovision). After testing was complete mice were removed and unburied marbles were counted. Marbles were considered buried if they were at least two-thirds covered by bedding. Number of marbles buried over 10 min time blocks and time spent digging/burying were scored manually with a stopwatch.

#### Self-grooming

Mice were placed in a clear plexiglass recording chamber with overhead video camera and self-grooming behavior was recorded for 30 min. Total time spent grooming was assessed over last 20 min of the recording.

#### Novel object recognition (NOR)

Mice were habituated to a square testing arena (30 × 30 cm) with visual cues on the walls, containing two identical objects (square Legos). Mice were allowed to freely explore these objects (familiar objects) for 10 min on 3 consecutive days (habituation). On Day 3, habituation was recorded using video tracking system (Ethovision) and time spent exploring each object was scored. Short-term memory (STM) test was carried out 1.5 h after habituation on Day 3. During STM test, the familiar object that each mouse spent less time exploring was replaced with a novel object (rectangular Lego). Long-term memory (LTM) test was carried out on Day 4 and the less explored object was once again replaced with another novel object (round Lego). Exploration was defined as contact with the object (nose point) or facing the object (distance < 2 cm). Time spent exploring the novel object was divided by the total time exploring both the novel and familiar objects to calculate a preference index (PI). A PI value of 0.5 indicates preference for neither object. A PI > 0.5 indicates a preference for the novel object, conversely a PI < 0.5 indicates a preference for the familiar object.

#### Object location memory (OLM) test

On Day 1, mice were habituated for 10 min to a square testing arena (30 × 30 cm) with visual cues on the walls (total 2 trials). On Day 2, two identical objects (square Legos) were placed equidistant from one wall and mice were allowed to freely explore the objects for 6 min (total 3 trials). On Day 3, one of the objects was moved to a different location and mice were allowed to explore the objects for 6 min while their activity was recorded with video tracking system (EthoVision). Exploration was defined as contact with the object (nose point) or facing the object (distance < 2 cm). Time spent exploring the object in a novel location was divided by the amount of time exploring both objects to calculate a PI (as in NOR test).

#### Three chamber social interaction (3CSI) test

A three-chamber arena with openings between the chambers was used to assess social preference and social novelty. The test mouse was placed in the center chamber at the start of each trial. A wire cage was placed in the left and right chambers. For habituation, test mice were placed in the center chamber and allowed to freely explore the arena and empty wire cages for 5 min. The social preference test began immediately after habituation. An unfamiliar mouse (stranger 1, male C57BL/6 J, age matched) was placed in one of the wire cages of the side chamber, while an object was placed in the other wire cage. The test mouse was again placed in the center chamber and allowed to explore the three-chamber arena for 5 min. After the social preference test, a new unfamiliar mouse (stranger 2, male C57BL/6 J, age matched) was placed in the wire cage that previously contained an object. The test mouse was allowed to explore the arena for 5 min to assess social novelty. The location of the object and stranger 1 was alternated between side chambers for different test mice to prevent chamber biases. The time spent in each chamber and time spent exploring the object, stranger 1 and stranger 2 were scored using video tracking software (EthoVision). Exploration was defined as contact with the wire cage containing the stranger or an object (nose point) or facing the wire cage (distance < 2 cm). Preference index (PI) was calculated as the amount of time spent exploring the wire cage with stranger 1 or stranger 2 divided by the total time spent exploring the wire cages during each trial.

#### Morris water maze

Mice were trained to find a hidden platform of a Morris water maze on four consecutive days. Training consisted of four trials on day 1 (60 s maximum, intertrial interval (ITI) 15 min) and three trials on each subsequent day. Visual cues were placed on the walls of the experimental room and on sides of the Morris water maze arena. A probe test was administered on day 5. During the probe test, the hidden platform was removed, and mice were allowed to swim in the arena for 1 min while their activity was recorded with overhead video tracking system (Ethovision). On days 6–8, the hidden platform was moved to a different quadrant and mice were trained to find the new location (reversal). A visible platform test was given one hour after final reversal trial. It consisted of four trials (ITI 15 min) with an escape platform marked by a visible cue (flag). Escape latency during training/reversal portions, and platform crossings/time spent in each quadrant during probe trial were scored using tracking software.

#### Y water maze

Mice were habituated to a water-based Y maze apparatus on day 1. On day 2, mice were trained to locate a submerged escape platform (hidden in water) in one of the arms of the Y maze until they made the correct arm choice (the one containing escape platform) on 4 consecutive trials (60 s maximum, ITI 15 min). Additional trials were given if mice did not reach this criterion. On day 3, a LTM test was administered to assess if mice achieved a success criterion of 4/5 correct consecutive trials. Mice that achieved this criterion underwent a reversal test 1.5 h later with the escape platform location switched to the other arm. Mice were trained to learn the new escape platform location with a success criterion of 9/10 correct arm choices on consecutive trials. Mice were not directed to the correct arm if they made an error in arm choice, rather, they were trapped in the arm for 20 s. Mice that met the 9/10 success criterion were not tested further.

#### Associative threat conditioning

On day 1, mice were placed in a clear Plexiglass training chamber and habituated to the testing apparatus for 15 min. On day 2, training for contextual and cued threat conditioning was performed, which consisted of a 150 s exploration period followed by two conditioned stimulus-unconditioned stimulus (CS-US) pairings separated by one min (foot-shock intensity 0.9 mA, 2 s duration; tone 85 db white noise, 30 s duration). Context and auditory cue tests were performed in the training chamber on day 3 (1.5 h apart). For the cue test, the training chamber was altered by placing white plexiglass with vanilla-scented bedding on top of the wire floor and changing white light to red. On days 4–5, CS extinction was carried out where each day consisted of 15 CS presentations during 22 min (in the same environment as the cue test). CS reactivation was tested on day 6 with the training chamber altered (textured rubber mat with a peppermint scent). Baseline freezing was monitored (3 min) prior to presentation of the tone and freezing was assessed during each portion of the test.

#### FUNCAT in slices

Acute hippocampal or cortical slices (400 µm thick) were prepared according to standard protocols. Slices were allowed to recover for 2 h in oxygenated ACSF and then azidohomoalanine (AHA, 1 mM) was added (AHA was omitted in ‘no AHA’ control condition). After 2.5 h incubation, slices were fixed overnight in 4% paraformaldehyde (PFA) in PBS. The following day, slices were embedded in agar and re-sectioned (30 μm), permeabilized and blocked in 5% normal goat serum/1% BSA in 0.1 M PBS for 2 h at RT. Click-it® reaction was performed using 1 μM Alexa Fluor-647 or Alexa Fluor-555 overnight at RT according to manufacturer’s protocol (ThermoFisher Scientific). Slices were washed and immunocytochemistry was performed using antibodies against parvalbumin, somatostatin and FMRP (see below). Following secondary antibody staining, slices were mounted and imaged using Leica LSM8 confocal microscope (40 × plan apochromat objective, NA = 1.4). Images were obtained using the same settings for all samples within an experiment. Experimenter was blind to the treatment condition. Fluorescence was quantified using ImageJ (NIH) in single image planes by generating an ROI around cell soma and selecting the image stack with greatest signal in AHA channel (DAPI was used to confirm presence of cell bodies). Background was subtracted using average values from ‘no AHA’ control condition. Slices prepared from pairs of WT (or WT-PV/WT-SOM) and KO (or cKO) mice were handled at the same time per individual experiment and treated in virtually identical manner throughout the experimental protocol. Subsequently, for each pair, fluorescence values obtained were normalized to mean fluorescence value of a matched WT control from the same experiment before pooling together values from different experiments/mice (so that fluorescence was expressed as fold change over WT for each individual experiment and then pooled together with other experiments).

#### Immunocytochemistry in slices

Mice were deeply anesthetized with ketamine and xylazine mixture (150 mg/kg and 15 mg/kg, respectively), and transcardially perfused with PBS followed by 4% PFA in PBS. Brains were dissected and fixed in 4% PFA for additional 24 h at 4ºC. Free-floating serial coronal sections (30 µm) were obtained using a Leica Vibratome. After blocking in 5% normal goat serum in 0.1 M PBS with 0.1% Triton X-100, brain sections were probed overnight with combinations of the following primary antibodies: mouse anti-parvalbumin (1:10,000, Sigma, P3088), rat anti-somatostatin (1:300, Millipore, MAB354), rabbit anti-FMRP (1:300, Sigma, F4055), mouse anti-FMRP (1:300, Biolegend, 834701) and rabbit anti-phospho-S6 ribosomal protein Ser235/236 (1:150, Cell Signaling, 4858). After primary antibody incubation, sections were washed three times in 0.1 M PBS, and subsequently incubated with Alexa Fluor conjugated secondary antibodies in blocking buffer for 1.5 h at room temperature. Sections were mounted using Prolong Gold antifade mounting media with DAPI for nuclear staining and imaged using Leica LSM8 confocal microscope. Fluorescence was quantified using ImageJ (NIH) in single image planes by drawing an ROI around a cell body (DAPI or NeuN was used to confirm the presence of cell bodies). Additional regions were sampled to obtain nonspecific background fluorescence and corrected total cell fluorescence (CTCF) was calculated using the following formula: CTCF = Integrated Density – (Area of selected cell X Mean fluorescence of background readings).

#### Data acquisition and statistical analysis

All behavioral experiments were performed in a blind manner. Sample size was chosen based on previous publications. Data were represented as mean ± SEM. All statistical analyses in this study were performed using GraphPad Prism software (version 8). For two-group comparisons, statistical significance was determined using parametric two-tailed Student’s t test. Multi-group analyses were done using one-way ANOVA, two-way ANOVA, or repeated measures (RM) two-way ANOVA (indicated for each experiment), followed by a Bonferroni multiple comparisons test. Extreme outliers were detected by applying the ROUT method (*Q* = 0.1%) and eliminated from further analysis (GraphPad Prism software). Statistical significance was reported as follows: *p < 0.05; **p < 0.01; ***p < 0.001; ns, not significant. *P* values < 0.05 were considered statistically significant.

## Results

### Deletion of *Fmr1* results in elevated de novo protein synthesis in somatostatin and parvalbumin inhibitory neurons

**Fig. 1:**
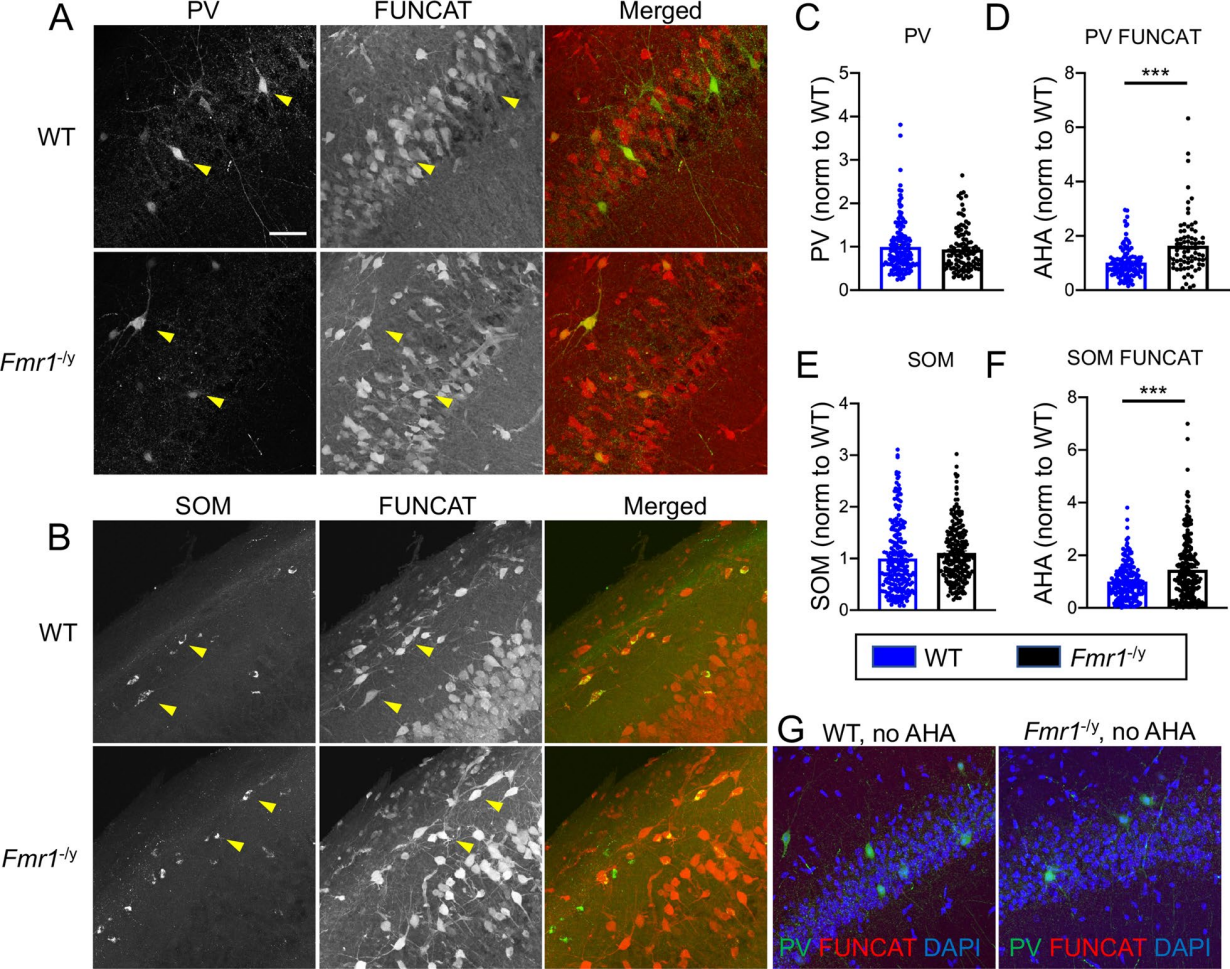
De novo protein synthesis is elevated in PV and SOM-positive neurons in *Fmr1*^−/y^ mouse hippocampus. **A–B** Representative images of FUNCAT in hippocampal slices from WT and *Fmr1*^−/y^ mice, co-stained for either PV **A** or SOM (**B**, scale bar: 50 μm). **C–D** Quantification of PV labeling **C** or AHA fluorescence **D** in PV-positive WT and *Fmr1*^−/y^ mouse hippocampal cells. Values represent mean ± SEM (*n* = 20–25 z-stacks, 9–11 sections, from 3 animals per genotype). **E–F** Quantification of SOM labeling **E** or AHA fluorescence **F** in SOM-positive WT and *Fmr1*^−/y^ mouse hippocampal cells. Values represent mean ± SEM (*n* = 26–39 z-stacks, 11–13 sections from 3 animals per genotype). **G** Representative images of FUNCAT control in WT and *Fmr1*^−^.^/y^ mouse hippocampal slices where AHA was omitted; ***p < 0.0001 (Student’s *t* test)

Elevated de novo protein synthesis is one of the core pathophysiological phenotypes reported in *Fmr1*^−/y^ mouse brain [11]. However, assessment of de novo protein synthesis in specific populations of inhibitory neurons has not been carried out in *Fmr1*^−/y^ mice [12]. To explore the role of FMRP in PV and SOM-positive inhibitory neurons, we first examined FMRP expression in these neuronal populations in the mouse hippocampus. We performed immunostaining for FMRP and PV or SOM in wild type (WT) mouse hippocampus and found that FMRP signal localized to both PV and SOM-expressing cells, whereas FMRP labeling was absent in these cells in the *Fmr1*^−/y^ mouse hippocampus (Additional file 1: Fig. S1). Next, to quantify the levels of de novo protein synthesis in PV and SOM-expressing neurons, we performed fluorescent non-canonical amino acid tagging (FUNCAT) along with immunostaining for PV and SOM in hippocampal slices from *Fmr1*^−/y^ and WT littermates (Fig. 1A, B). The levels of PV and SOM expression were not changed in *Fmr1*^−/y^ compared to WT mouse hippocampus (Fig. 1C , *p* > 0.1 and Fig. 1E, *p* = 0.059, Student’s t test, NS). However, the FUNCAT signal was significantly elevated in *Fmr1*^−/y^ mouse PV and SOM-positive neurons in the hippocampus compared to WT (Fig. 1D, ***p < 0.0001 and Fig. 1F, ***p < 0.0001, Student’s t test) and was near background levels in *Fmr1*^−/y^ and WT hippocampal slices where azidohomoalanine (AHA) was omitted (Fig. 1G). Thus, consistent with previous studies of several brain regions of the *Fmr1*^−/y^ mouse [11, 12, 27], de novo protein synthesis was increased in PV and SOM-positive neurons in the hippocampus of *Fmr1*^−/y^ mice.

### Mice with cell-type specific deletion of *Fmr1* in PV-positive neurons exhibit mild anxiety-like behaviors and impaired social interaction

**Fig. 2:**
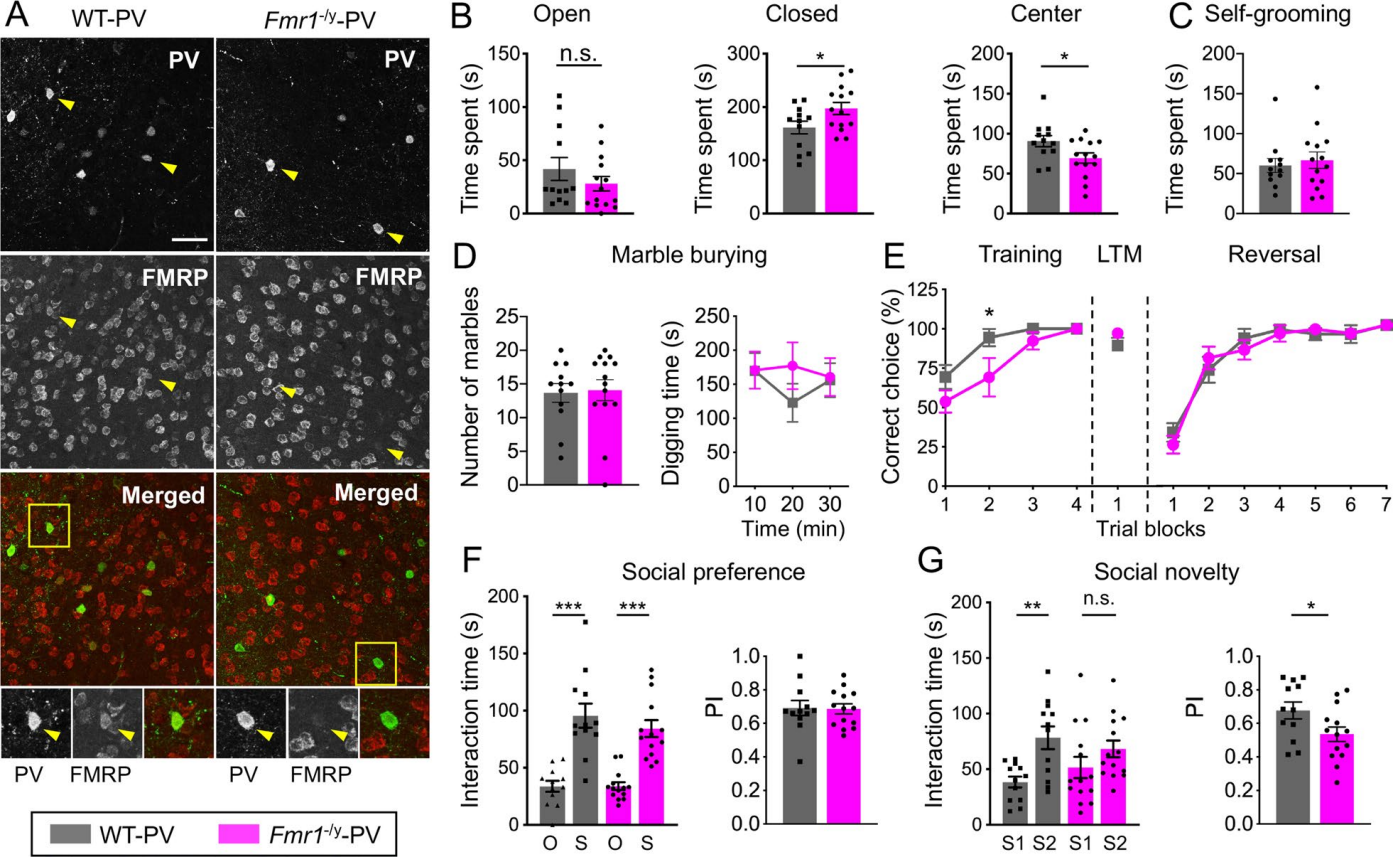
Mice lacking *Fmr1* in PV-expressing neurons exhibit mild anxiety-like behaviors and impaired social interaction. **A** Representative images of mPFC sections from WT-PV and *Fmr1*^−/y^-PV mice, immunostained for PV and FMRP (scale bar: 50 μm). Bottom panels show enlarged boxed regions of individual PV-positive cells. **B** Time spent in the open arms of EPM was not significantly different between the genotypes (left), whereas *Fmr1*^−/y^-PV mice spent more time in the closed arms (middle) and less time in the center (left) of the EPM; mean ± SEM (*n* = 12–14 animals per genotype). **C** Time spent self-grooming and **D** total number of marbles buried, as well as time spent burying/digging up marbles did not differ significantly between WT-PV and *Fmr1*^−/y^-PV mice. Values represent mean ± SEM (*n* = 10–14 animals per genotype). **E** *Fmr1*^−/y^-PV mice made significantly greater percentage of errors during the training portion of the Y Maze task, whereas their performance during LTM test and reversal portions was not significantly different between the genotypes. Values represent mean ± SEM (*n* = 12–13 animals per genotype). **F** *Fmr1*^−/y^-PV and WT-PV mice spent more time sniffing a stranger mouse (*S*) compared to an object (*O*) during Sociability portion of the 3CSI Test. Values represent mean ± SEM (*n* = 12–14 animals per genotype). Preference Index (PI) for a stranger mouse was not different between the genotypes. **G** WT-PV mice spent significantly more time sniffing a novel stranger mouse (*S*2) compared to a familiar stranger mouse (*S*1) during the Social Novelty test, while *Fmr1*^−^.^/y^-PV mice exhibited impaired social novelty behavior. (*n* = 12–14 animals per genotype). PI for a S2 was also significantly different between the genotypes; *p < 0.05; **p < 0.01; ***p < 0.001; Two-way ANOVA or RM two-way ANOVA followed by Bonferroni’s multiple comparisons test, Student’s *t* test

To determine the cell type-specific contribution of FMRP expression in PV-positive neurons to autistic-like behavioral deficits, we utilized Cre-lox technology to generate mice with a cell type-specific deletion of *Fmr1* in PV-expressing neurons, termed *Fmr1*^flx/flx^:Pv-Cre (*Fmr1*^−/y^-PV) mice. First, we confirmed the lack of FMRP expression in PV-positive neurons in *Fmr1*^−/y^-PV mouse mPFC (Fig. 2A). We found that approximately 49% and 59% of PV-positive cells expressed FMRP in WT-PV mouse mPFC and hippocampus, respectively. We quantified FMRP fluorescence in all PV-expressing cells in WT-PV and *Fmr1*^−/y^-PV mice and observed a significant reduction of FMRP expression in *Fmr1*^−/y^-PV mPFC and hippocampus (Additional file 1: Fig. S2A, ***p < 0.0001 and B, ***p < 0.0001, Student’s *t* test). We believe the residual fluorescent signal observed in *Fmr1*^−/y^-PV was due to nonspecific FMRP antibody signal in FMRP negative cells (see staining in *Fmr1*^−/y^ mice, Additional file 1: Fig. S1, bottom panel). Next, we performed a battery of tests to assess motor function, anxiety, repetitive, stereotypic, and social behaviors in Fmr1^+/+^:Pv-Cre (WT-PV) and *Fmr1*^−/y^-PV mice. *Fmr1*^−/y^-PV mice displayed normal motor behavior and learning (Additional file 1: Fig. S2C, two-way RM ANOVA, followed by Bonferroni’s multiple comparisons test; trial x genotype, Day 1, F(3, 72) = 0.599, *p* > 0.1; Day 2, F(3, 72) = 1.552, *p* > 0.1). Using elevated plus maze (EPM), we assessed anxiety-like behavior in WT-PV and *Fmr1*^−/y^-PV mice. Time spent in the open arms of EPM was not different between the genotypes (mean ± SEM: WT-PV 41.81 s ± 10.71, *Fmr1*^−/y^-PV 28.04 s ± 6.75, *p* > 0.1, Student’s *t* test), but *Fmr1*^−/y^-PV mice spent significantly more time than WT-PV mice in the closed arms (mean ± SEM: WT-PV 161.5 s ± 11.98, *Fmr1*^−/y^-PV 197.3 s ± 11.40, *p = 0.041, Student’s *t* test) but significantly less time in the center (mean ± SEM: WT-PV 90.60 s ± 7.05, *Fmr1*^−/y^-PV 69.48 s ± 6.38, *p = 0.036, Student’s *t* test) of the EPM (Fig. 2B), indicative of elevated anxiety.

We used self-grooming and marble bury tests to assess if *Fmr1*^−/y^-PV mice exhibit repetitive and stereotypic behaviors, one of the criteria for ASD diagnosis [28]. Time spent self-grooming (mean ± SEM: WT-PV 60.15 s ± 8.75, *Fmr1*^−/y^-PV 66.77 s ± 10.24, *p* > 0.1, Student’s *t* test), the total number of marbles buried (mean ± SEM: WT-PV 13.67 ± 1.38, *Fmr1*^−/y^-PV 14.07 ± 1.57, *p* > 0.1, Student’s *t* test), or time spent burying/digging up marbles (two-way RM ANOVA, followed by Bonferroni’s multiple comparisons test; time x genotype, F(2, 42) = 1.389, *p* > 0.1) was not significantly different between the genotypes (Fig. 2C, D). Thus, *Fmr1*^−/y^-PV mice do not exhibit these forms of repetitive behaviors. Rigidity in behavior and the need for sameness are manifestations of perseverative behaviors associated with ASD [28]. We used a water-based Y-maze task to assess cognitive flexibility in mice with cell type-specific deletion of *Fmr1*. *Fmr1*^−/y^-PV mice were impaired during initial acquisition portion of the task as evident by a significant decrease in percentage of correct arm choices during training (Fig. 2E, training portion, two-way RM ANOVA, followed by Bonferroni’s multiple comparisons test; time x genotype, F(3, 69) = 1.977, *p* > 0.1; a significant difference between genotypes during trial block 2 using post-hoc Bonferroni’s multiple comparisons test, *p = 0.027). However, after a criterion level of performance was achieved, *Fmr1*^−/y^-PV mice performed similar to WT-PV mice during long-term memory (LTM) testing and reversal portions of the test (Fig. 2E, LTM, *p* > 0.1, Student’s t test, NS; reversal, two-way RM ANOVA, followed by Bonferroni’s multiple comparisons test; time x genotype, F(6, 138) = 0.583, *p* > 0.1). Therefore, mice with *Fmr1* deletion in PV neurons exhibit normal cognitive flexibility.

Deficits in social interaction represent another group of diagnostic criteria associated with ASD [28]. In the sociability portion of the 3-chamber social interaction (3CSI) test, both *Fmr1*^−/y^-PV and WT-PV mice spent more time exploring and sniffing a novel mouse (S) compared to an object (O), Fig. 2F, two-way ANOVA, followed by Bonferroni’s multiple comparisons test, F(1, 48) = 64.74, ***p < 0.0001). In addition, there was no significant difference between the genotypes in the preference index (PI) for the novel mouse, indicating normal social preference behavior in *Fmr1*^−/y^-PV mice (Fig. 2F, *p* > 0.1, Student’s *t* test). On the other hand, during the social novelty portion of the test, WT-PV mice displayed a natural preference to spend more time exploring/sniffing a novel mouse (stranger 2, S2), whereas *Fmr1*^−/y^-PV did not show a preference for interacting with S2 (Fig. 2G, two-way ANOVA, followed by Bonferroni’s multiple comparisons test, F(1, 48) = 11.34, **p < 0.01, *p* > 0.1). Furthermore, *Fmr1*^−/y^-PV had a significantly decreased PI for S2, suggesting they did not exhibit a preference for social novelty (Fig. 2G, *p* = 0.041, Student’s t test). This was unlikely due to their mild anxiety, as the total time spent interacting with *S*1 + *S*2 during social novelty test did not differ between the genotypes (mean total interaction time ± SEM: WT-PV 116.8 s ± 9.98, *Fmr1*^−/y^-PV 119.8 s ± 10.22, *p* = 0.831, Student’s *t* test). Taken together, these findings suggest that ablation of FMRP expression in PV-positive neurons results in a specific pattern of ASD-like behavioral deficits (Table 1).

**Table 1:** Summary of behavioral alterations in *Fmr1*^−/y^-PV and *Fmr1*^−^.^/y^-SOM mice compared to control

Phenotype	Test	*Fmr1*^−/y^-PV	*Fmr1*^−/y^-SOM
Motor function and learning	Rotarod		Not shown (no deficit)
Anxiety-like behavior	Elevated plus maze	Increased time in closed arms and decreased time in the center	–
ASD-like behavior	Marble burying	–	–
	Self-grooming	–	–
	Y Water Maze	Increased errors during training	–
	Sociability and social novelty	Deficit in social novelty behavior	–
Learning and memory	Novel object recognition	–	–
	Object location memory	–	Not done
	Morris water maze	–	–
	Threat conditioning	Increased freezing during tone presentation (CS testing and reconsolidation)	–

### *Fmr1*^*−/y*^-PV mice do not display deficits in learning and memory

**Fig. 3:**
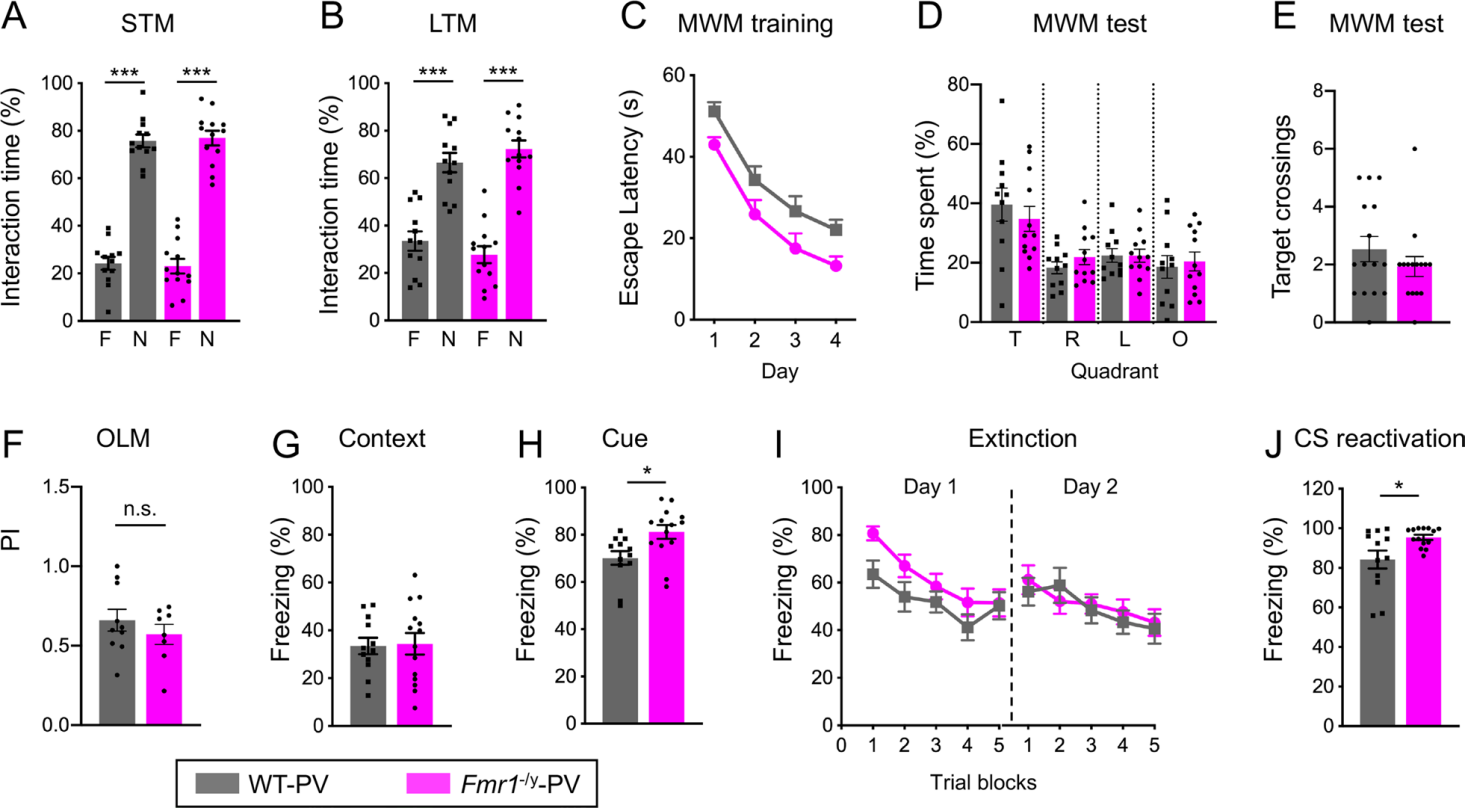
Mice with *Fmr1* deletion in PV-positive neurons do not display deficits in learning and memory. **A**, **B** *Fmr1*^−/y^-PV and WT-PV mice spent more time exploring a novel object during STM **A** and LTM **B** portions of the object recognition test. Values represent mean ± SEM (*n* = 12–13 animals per genotype). **C** Escape latency during the training portion of MWM was not significantly different between *Fmr1*^−/y^-PV and WT-PV mice. Values represent mean ± SEM (*n* = 15 animals per genotype). **D**, **E** Target quadrant occupancy **D** or frequency of platform crossings during 60 s probe trial of MWM test were not significantly different between *Fmr1*^−/y^-PV and WT-PV mice. Values represent mean ± SEM (*n* = 15 animals per genotype). **F** Preference index for a novel object location during OLM test was not significantly different between *Fmr1*^−/y^-PV and WT-PV mice. Values represent mean ± SEM (*n* = 8–10 animals per genotype). **G**, **H** Following threat conditioning, *Fmr1*^−/y^-PV mice spent similar percentage of time freezing to the context **G** but spent significantly more time freezing to the audible cue **H** compared to WT-PV mice. Values represent mean ± SEM (*n* = 12–14 animals per genotype). **I** Acquisition of threat memory extinction was not significantly different between the genotypes. Freezing to the tone shown in trial blocks on day 1 and day 2 of threat memory extinction. Values represent mean ± SEM (*n* = 12–14 animals per genotype). **J** *Fmr1*^−/y^-PV mice spent significantly more time freezing to the audible cue following reactivation of auditory threat memory. Values represent mean ± SEM (*n* = 12–14 animals per genotype); ***p < 0.001; *p < 0.05; Two-way ANOVA or RM two-way ANOVA followed by Bonferroni’s multiple comparisons test, Student’s *t* test

Intellectual disability is a prominent feature of FXS affecting over 85% of males and 25% of females to varying levels of severity [4, 5]. To examine the impact of *Fmr1* deletion in PV positive neurons on learning and memory, we carried out a series of cognitive tasks with the *Fmr1*^−/y^-PV and WT mice. During the object recognition task, *Fmr1*^−/y^-PV and WT-PV mice showed a preference for the novel object during both short-term (STM) (two-way ANOVA, followed by Bonferroni’s multiple comparisons test, F(1, 46) = 324.2, ***p < 0.0001) and long-term memory (LTM) (two-way ANOVA, followed by Bonferroni’s multiple comparisons test, F(1, 46) = 102.0, ***p < 0.0001) portions of the test (Fig. 3A, B). In addition, PI for novel object during STM and LTM was not different, indicating intact memory formation and recall (Additional file 1: Fig. S3A, *p* > 0.1, Student’s t test).

We next tested spatial memory in *Fmr1*^−/y^-PV and WT mice using Morris Water Maze (MWM). Escape latency during training did not differ significantly between the genotypes (Fig. 3C, two-way RM ANOVA, followed by Bonferroni’s multiple comparisons test; time x genotype, F(3, 84) = 0.017, *p* > 0.1). On the probe test day, *Fmr1*^−/y^-PV mice spent more time in the target quadrant of the maze, similar to WT-PV mice (Fig. 3D, two-way RM ANOVA, followed by Bonferroni’s multiple comparisons test, quadrant x genotype, F(3, 84) = 0.674, *p* > 0.1). Even though *Fmr1*^−/y^-PV mice showed a trend for reduced platform crossings during the probe test, it did not reach significance (Fig. 3E, *p* > 0.1, Student’s t test). In addition, escape latency during MWM reversal portion was not significantly different between the genotypes (Additional file 1: Fig. S3B, two-way RM ANOVA, followed by Bonferroni’s multiple comparisons test, time x genotype, F(2, 56) = 1.473, *p* > 0.1). *Fmr1*^−/y^-PV mice did not exhibit vision impairments that could affect their performance during MWM, as there was no difference in escape latency between the genotypes when the platform was marked by a visible cue (Additional file 1: Fig. S3C, *p* > 0.1, Student’s t test). To further discern whether *Fmr1*^−/y^-PV mice exhibit deficits in hippocampus-dependent spatial memory, we used the object location memory (OLM) task. PI for the object in a novel versus familiar location did not differ between *Fmr1*^−/y^-PV and WT-PV mice, indicating intact hippocampal memory (Fig. 3F, *p* > 0.1, Student’s t test). Lastly, we performed a threat conditioning task to test the associative memory of *Fmr1*^−/y^-PV and WT-PV mice. *Fmr1*^−/y^-PV mice spent similar percentage of time freezing to the context associated with the shocks 24 h after training (Fig. 3G, *p* > 0.1, Student’s t test) but spent significantly more time freezing during tone presentation compared to WT-PV mice (Fig. 3H, *p* = 0.014, Student’s t test). Acquisition of threat memory extinction was not significantly different between the genotypes (Fig. 3I, two-way RM ANOVA, followed by Bonferroni’s multiple comparisons test, time block x genotype, Day 1, F(4, 96) = 1.849, *p* > 0.1; Day 2, F(4, 96) = 1.050, *p* > 0.1). In addition, *Fmr1*^−/y^-PV mice spent significantly more time freezing during tone presentation compared to WT-PV during reconsolidation portion of threat conditioning test (Fig. 3J, *p* = 0.018, Student’s t test). Together, these findings indicate that absence of FMRP in PV positive neurons is not associated with any significant deficits in learning and memory but may play a role in limiting long-term auditory threat memory and memory reconsolidation (Table 1).

### Deletion of *Fmr1* in SOM-positive neurons does not result in autistic-like or cognitive behavioral deficits

**Fig. 4:**
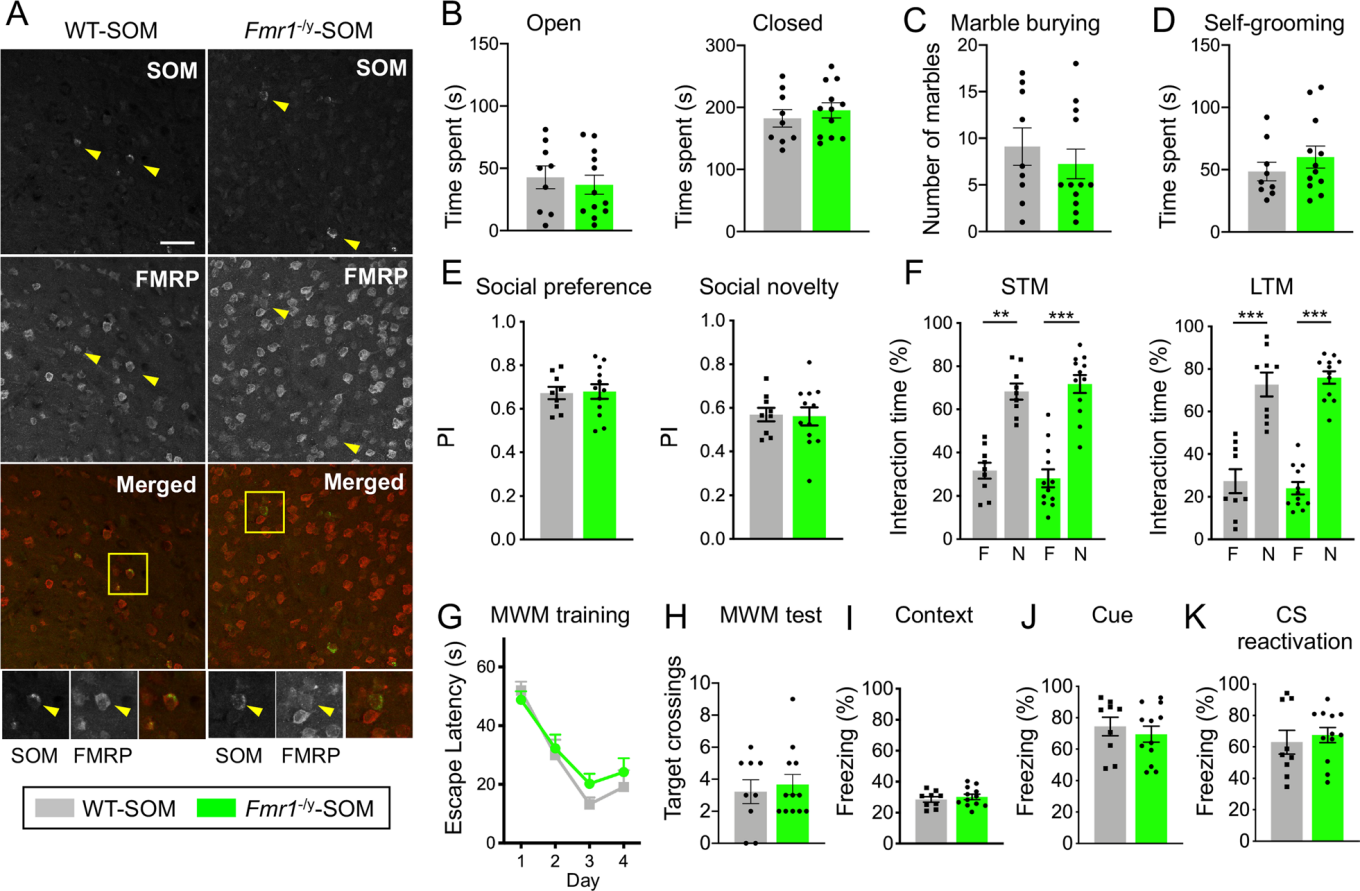
*Fmr1* deletion in SOM-expressing neurons does not result in cognitive or autistic-like deficits in mice. **A** Representative images of mPFC sections from WT-SOM and *Fmr1*^−/y^-SOM mice, immunostained for SOM and FMRP (scale bar: 50 μm). Bottom panels show enlarged boxed regions of individual SOM-positive cells in WT-SOM and *Fmr1*^−/y^-SOM mouse mPFC. **B** Time spent in the open or closed arms of the EPM, **C** number of marbles buried or **D** time spent self-grooming were not significantly different between WT-SOM and *Fmr1*^−/y^-SOM mice. Values represent mean ± SEM (*n* = 9–12 animals per genotype). **E** Preference Index (PI) for a stranger mouse (*S*) during Social Preference or a novel stranger mouse (*S*2) during Social Novelty portion were not significantly different between the genotypes. Values represent mean ± SEM (*n* = 9–12 animals per genotype). **F** *Fmr1*^−/y^-SOM and WT-SOM mice spent more time exploring a novel object during STM and LTM portions of the object recognition test. Values represent mean ± SEM (*n* = 9–12 animals per genotype). **G**, **H** Escape latency during the training portion of MWM **G** or frequency of platform crossings during 60 s probe trial **H** were not significantly different between *Fmr1*^−/y^-SOM and WT-SOM mice. Values represent mean ± SEM (*n* = 9–12 animals per genotype). **I–K** *Fmr1*^−^.^/y^-SOM mice spent similar percentage of time compared to WT-SOM mice in freezing behavior in the context associated with the shocks (I, contextual threat memory), as well as during tone presentation (J, cued threat memory). In addition, there was no significant difference between the genotypes in time spent freezing to the audible cue following reactivation of auditory threat memory (**K**). Values represent mean ± SEM (*n* = 9–12 animals per genotype); ***p < 0.001; *p < 0.05; Two-way ANOVA or RM two-way ANOVA followed by Bonferroni’s multiple comparisons test, Student’s *t* test

As PV- and SOM-positive inhibitory neurons play different roles in circuit function and behavior, we wanted to dissect the cell type-specific role of FMRP in SOM-expressing neurons in autism-associated behaviors and cognition. To this end, we generated mice with *Fmr1* deletion in SOM-expressing neurons, the Fmr1^flx/flx^: Sst-IRES-Cre (*Fmr1*^−/y^-SOM mice) and performed behavioral testing with the *Fmr1*^−/y^-SOM and Fmr1^+/+^: Sst-IRES-Cre (WT-SOM) mice. First, the lack of FMRP expression in SOM-positive inhibitory neurons was confirmed in *Fmr1*^−/y^-SOM mouse mPFC (Fig. 4A). Approximately 67% and 50% of SOM-positive cells expressed FMRP in WT-SOM mouse mPFC and hippocampus, respectively. FMRP signal was quantified in all SOM-expressing cells in WT-SOM and *Fmr1*^−/y^-SOM mice and a significant reduction of FMRP expression was observed in *Fmr1*^−/y^-SOM mPFC and hippocampus (Additional file 1: Fig. S4A, ***p < 0.0001 and 4B, ***p = 0.0001, Student’s t test). Once again, there was residual fluorescent signal observed in *Fmr1*^−/y^-PV due to nonspecific FMRP antibody signal in FMRP negative cells (see staining in in *Fmr1*^−/y^ mice, Additional file 1: Fig. S1, bottom panel). We proceeded to perform behavioral experiments and observed no difference in the amount of time spent in the open or closed arms of the EPM between WT-SOM and *Fmr1*^−/y^-SOM mice (Fig. 4B, time spent in the open arm, mean ± SEM: WT-SOM 42.77 s ± 9.23, *Fmr1*^−/y^-SOM 36.81 s ± 7.64, *p* > 0.1; time spent in the closed arm, mean ± SEM: WT-SOM 182.6 s ± 14.08, *Fmr1*^−/y^-SOM 195.4 s ± 12.16, *p* > 0.1, Student’s *t* test), indicating that *Fmr1*^−/y^-SOM mice do not exhibit anxiety-like behavior. In addition, *Fmr1*^−/y^-SOM mice did not exhibit repetitive, stereotypic behaviors as the number of marbles buried (mean ± SEM: WT-SOM 9.11 ± 2.00, *Fmr1*^−/y^-SOM 7.25 ± 1.59, *p* > 0.1, Student’s *t* test) and the time spent self-grooming (mean ± SEM: WT-SOM 48.52 s ± 7.43, *Fmr1*^−/y^-SOM 60.15 s ± 8.83, *p* > 0.1, Student’s *t* test) were not significantly altered compared to WT-SOM mice (Fig. 4C, D, respectively). In addition, we did not observe perseverative behavior in *Fmr1*^−/y^-SOM mice using the water-based Y-maze as percentage of correct arm choices was not different during training, LTM test or reversal portions of the test (Additional file 1: Fig. S4C, training portion, two-way RM ANOVA, followed by Bonferroni’s multiple comparisons test; time x genotype, F(3, 57) = 1.409, *p* > 0.1; LTM, *p* > 0.1, Student’s t test, NS; reversal portion, two-way RM ANOVA, followed by Bonferroni’s multiple comparisons test; time x genotype, F(6, 114) = 0.744, *p* > 0.1). Overall, these data indicate that *Fmr1*^−/y^-SOM mice did not exhibit anxiety-like, stereotypic, or perseverative behaviors.

In the 3CSI test, both WT-SOM and *Fmr1*^−/y^-SOM mice spent longer time sniffing and exploring novel S mouse over an object (Additional file 1: Fig. S4D, two-way ANOVA, followed by Bonferroni’s multiple comparisons test, F(1, 38) = 36.19, ***p < 0.0001), and PI for S was not different between the genotypes (Fig. 4E, *p* > 0.1, Student’s t test), indicating that *Fmr1*^−/y^-SOM mice exhibit normal social preference behavior. In the social novelty portion of the 3CSI, there was an overall trend for WT-SOM and *Fmr1*^−/y^-SOM mice to spend more time sniffing S2, however it was not significant (Additional file 1: Fig. S4E, two-way ANOVA, followed by Bonferroni’s multiple comparisons test, F(1, 38) = 2.901, *p* > 0.1). In addition, PI for S2 was at or below 0.5 (no preference) for 3/9 of WT-SOM mice and 6/12 of *Fmr1*^−/y^-SOM mice (Fig. 4E, *p* > 0.1, Student’s t test). Thus, overall social behavior was unaffected in *Fmr1*^−/y^-SOM mice compared to WT-SOM, but both genotypes displayed decreased preference for social novelty.

Assessment of learning and memory was performed in WT-SOM and *Fmr1*^−/y^-SOM mice. During the object recognition task, WT-SOM and *Fmr1*^−/y^-SOM mice spent longer time exploring a novel compared to familiar object during STM and LTM (Fig. 4F, STM two-way ANOVA, followed by Bonferroni’s multiple comparisons test, F(1, 19) = 48.12, ***p < 0.0001, **p < 0.001; LTM two-way ANOVA, followed by Bonferroni’s multiple comparisons test, F(1, 38) = 135.8, ***p < 0.0001). PI for novel object was also unchanged in *Fmr1*^−/y^-SOM mice (Additional file 1: Fig. S4F and G, *p* > 0.1, Student’s t test). Spatial memory in MWM was unaltered in *Fmr1*^−/y^-SOM mice; escape latency during training was similar between WT-SOM and *Fmr1*^−/y^-SOM mice (Fig. 4G, two-way RM ANOVA, followed by Bonferroni’s multiple comparisons test; time x genotype, F(3, 57) = 0.622, *p* > 0.1), as well as time spent in the target quadrant (Additional file 1: Fig. S4H, two-way RM ANOVA, followed by Bonferroni’s multiple comparisons test, quadrant x genotype, F(3, 57) = 0.086, *p* > 0.1) and number of platform crossings (*p* > 0.1, Student’s t test) on probe test day (Fig. 4H). Escape latency during MWM reversal (two-way RM ANOVA, followed by Bonferroni’s multiple comparisons test, time x genotype, F(2, 38) = 0.017, *p* > 0.1) and visible platform test (*p* > 0.1, Student’s t test) also did not differ between the genotypes (Additional file 1: Fig. S4I, J). Lastly, *Fmr1*^−/y^-SOM mice were tested in threat conditioning task and percentage of time spent in freezing behavior in the context associated with shocks and during tone presentation (Fig. 4I, J, respectively, *p* > 0.1, Student’s t test) or during reactivation of auditory threat memory were not different from WT-SOM (Fig. 4K, *p* > 0.1, Student’s t test). Overall, we did not observe significant autism-related or cognitive deficits in *Fmr1*^−/y^-SOM mice (Table 1).

### Protein synthesis is dysregulated in PV, but not SOM-positive neurons following cell type-specific deletion of Fmr1

**Fig. 5:**
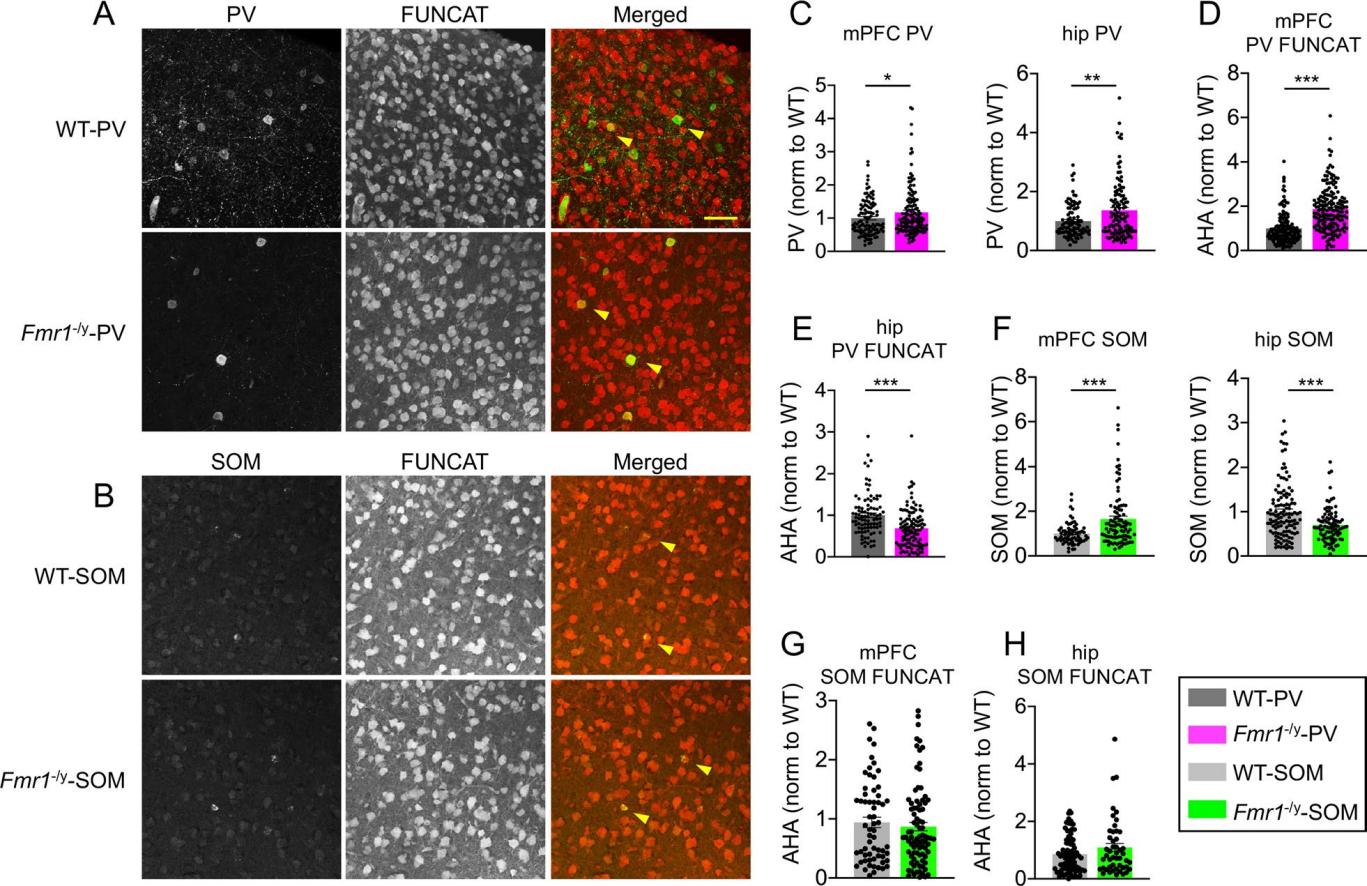
Brain region-specific cellular deficits in PV- and SOM-positive neurons upon cell type-specific deletion of *Fmr1.* **A**, **B** Representative images of FUNCAT and PV staining from WT-PV and *Fmr1*^−/y^-PV mouse mPFC (**A**, scale bar: 50 μm) or FUNCAT and SOM staining from WT-SOM and *Fmr1*^−/y^-SOM mice (**B**). Arrows denote individual PV- or SOM-expressing cells with FUNCAT signal. **C** PV expression was elevated in mPFC and hippocampus of *Fmr1*^−/y^-PV mouse. Quantification of PV labeling in PV-positive neurons in WT-PV and *Fmr1*^−/y^-PV mouse mPFC and hippocampus. **D**, **E** Cell type-specific deletion of *Fmr1* results in elevated de novo protein synthesis in PV-positive neurons in mPFC, but a decrease in hippocampus of *Fmr1*^−/y^-PV mice. Quantification of AHA fluorescence in PV-positive neurons in WT-PV and *Fmr1*^−/y^-PV mouse mPFC **D** and hippocampus **E**. Values represent mean ± SEM (hippocampus, *n* = 45–48 z-stacks, 19–24 sections, from 3 animals per genotype; mPFC, *n* = 24–25 z-stacks, 14 sections, from 3 animals per genotype); ***p < 0.0001 (Student’s *t* test). **F** SOM expression was elevated in mPFC but decreased in hippocampus of *Fmr1*^−/y^-SOM mice. Quantification of SOM labeling in SOM-positive neurons in WT-SOM and *Fmr1*^−/y^-SOM mouse mPFC and hippocampus. **G**, **H** De novo protein synthesis is not significantly different upon cell type-specific deletion of *Fmr1* in SOM-positive neurons. Quantification of AHA fluorescence in SOM-positive neurons in WT-SOM and *Fmr1*^−^.^/y^-SOM mouse mPFC **G** and hippocampus (**H**). Values represent mean ± SEM (hippocampus, *n* = 26–34 z-stacks, 11–14 sections, from 2 animals per genotype; mPFC, *n* = 39 z-stacks, 21–22 sections, from 3 animals per genotype); ***p < 0.0001 (Student’s *t* test)

To elucidate molecular alterations arising from cell type-specific ablation of FMRP, we examined PV and SOM expression and de novo protein synthesis using immunostaining and FUNCAT in mPFC and hippocampus (Fig. 5A, B), forebrain regions relevant to the behaviors examined in Figs. 2, 3, and 4. We found that expression of PV itself was elevated both in *Fmr1*^−/y^-PV hippocampus and mPFC compared to WT-PV (Fig. 5C, hippocampus **p < 0.01; mPFC; *p < 0.05, Student’s t test). Furthermore, we found that de novo protein synthesis was elevated in PV-positive neurons in *Fmr1*^−/y^-PV mPFC but decreased in the hippocampus (Fig. 5D, E, respectively, hippocampus ***p < 0.001; mPFC ***p < 0.001, Student’s t test). To further characterize brain region-specific cellular deficits in regulation of protein synthesis by FMRP in PV-expressing neurons, we examined phosphorylation of ribosomal protein S6 (rpS6), a downstream effector of mammalian target of rapamycin complex 1 (mTORC1), in *Fmr1*^−/y^-PV mPFC and hippocampus. Signaling by mTORC1 leads to activation of protein synthesis and both processes are elevated in *Fmr1*^−/y^ mice [27, 29]. Importantly, phosphorylation of rpS6 was also enhanced in *Fmr1*^−/y^ mouse hippocampal neurons [15]. Our results indicate that rpS6 phosphorylation (Ser 235/6) was increased in PV-positive hippocampal neurons but unaltered in PV-positive inhibitory neurons in mPFC of *Fmr1*^−/y^-PV mice (Additional file 1: Fig. S5, hippocampus *p < 0.016; mPFC *p* > 0.1, Student’s t test). Thus, there is a brain region-specific dysregulation of de novo protein synthesis and mTORC1 downstream signaling upon cell type-specific deletion of *Fmr1* in PV-positive inhibitory neurons, which may underlie the specific behavioral deficits (Additional file 2: Table S1).

Expression of SOM was significantly increased in mPFC but decreased in the hippocampus of *Fmr1*^−/y^-SOM (Fig. 5F, hippocampus *** *p* < 0.001; mPFC ***p < 0.001, Student’s t test). On the other hand, de novo protein synthesis in SOM-positive inhibitory neurons did not differ between WT-SOM and *Fmr1*^−/y^-SOM mice, both in mPFC and hippocampus (Fig. 5G, H, respectively, hippocampus *p* > 0.1; mPFC; *p* > 0.1, Student’s t test). Similar to findings from behavioral experiments, global de novo protein synthesis was unaffected by cell type-specific deletion of *Fmr1* in SOM-expressing inhibitory neurons. Together, these findings indicate that PV neurons appear to be particularly sensitive to FMRP ablation at the molecular and behavioral level (Additional file 2: Table S1).

## Discussion

Our data indicate that ablating FMRP expression in PV-positive neurons recapitulates some of the behavioral deficits associated with FXS, including impaired social interaction and elevated anxiety, but does not result in impairments in learning and memory. Furthermore, we found that *Fmr1* deletion in PV-positive neurons resulted in brain region-specific dysregulation of de novo protein synthesis in PV-positive cells whereas deletion of *Fmr1* in SOM-expressing inhibitory neurons did not result in any behavioral deficits and no effect on global de novo protein synthesis was observed in these cells. Our findings suggest a differential role for FMRP expression in two major inhibitory neuron populations and increase our understanding of the cell type-specific mechanisms underlying FXS phenotypes. As FMRP is ubiquitously expressed in cells and present in most brain regions [30–33], selective deletion in specific cell types likely will result in a particular pattern of FXS-like phenotypes. The contribution of FMRP expression in different CNS cell types to FXS pathophysiology is now beginning to be elucidated, but the effects of *Fmr1* deletion in GABAergic neurons have not been previously described. Selective deletion of *Fmr1* from forebrain excitatory neurons induces cellular, electrophysiological, and behavioral phenotypes in mice including increased mTOR/Akt phosphorylation and enhanced locomotor activity, but not anxiety-like behavior [34]. In addition, mice with deletion of *Fmr1* in forebrain excitatory neurons exhibit deficits in PV-positive neuron density and perineuronal net formation. FMRP ablation in astroglial cells results in cellular, synaptic, and behavioral deficits, including elevated protein synthesis, increased neuronal spine density, enhanced phosphorylation of rpS6, and impaired learning of a motor task [35, 36].

*Fmr1*^−/y^ mice exhibit several autistic-like behaviors and cognitive impairments, although specific findings vary between different labs, which has been attributed to differences in the mouse genetic background [37]. *Fmr1*^−/y^-PV mice recapitulate some of the behavioral deficits previously described in *Fmr1*^−/y^. Hyperactivity has been reported in *Fmr1*^−/y^ mice [38, 39], although we did not observe hyperactivity in *Fmr1*^−/y^-PV mice (data not shown). No changes in anxiety-like behavior or reduced anxiety have both been reported in *Fmr1*^−/y^ mice [26, 39], which contrasts with elevated anxiety we observed in *Fmr1*^−/y^-PV mice (Fig. 2B). Furthermore, *Fmr1*^−/y^ mice exhibit stereotypic and repetitive behaviors in the marble burying task and increased self-grooming [13, 26]. We did not observe these behaviors in *Fmr1*^−/y^-PV mice (Fig. 2C, D). In addition, *Fmr1*^−/y^ mice exhibit perseverative behaviors during water Y maze reversal [15] whereas we observed deficit during training but not reversal portion of the Y maze in *Fmr1*^−/y^-PV mice (Fig. 2E). Impairments in communication (reduced number of ultrasonic vocalizations) and deficits in social interaction have been reported in *Fmr1*^−/y^ mice [13, 40]. Social vocalizations were not tested in *Fmr1*^−/y^-PV mice. We observed impaired social novelty behavior in *Fmr1*^−/y^-PV (Fig. 2G) which is in line with findings in *Fmr1*^−/y^ [13]. Deficits in learning and memory have also been reported in *Fmr1*^−/y^ mice. During MWM, fewer platform crossings in MWM on probe trial day, slight deficit in training or no deficits have been reported in *Fmr1*^−/y^ mice [26, 41, 42]. We did not observe significant deficits in *Fmr1*^−/y^-PV mice during MWM (Fig. 2C, D and E). Furthermore, no deficits in threat conditioning or impaired cue and context freezing were reported in *Fmr1*^−/y^ mice [26, 41, 43]. We did not observe deficits in threat conditioning in *Fmr1*^−/y^-PV mice, but we observed enhanced freezing during auditory tone presentation (Fig. 3G–J). Thus, cell-type specific deletion of *Fmr1* in PV + neurons recapitulates some, but not all, of the behavioral deficits described in global *Fmr1*^−/y^ mice.

Elevated or dysregulated neuronal protein synthesis has been postulated as one of the key mechanisms underlying development of ASD and it has been demonstrated that manipulating expression of proteins controlling cap-dependent translation initiation, such as 4E-BP and eIF4E, results in aberrant, autistic-like behavioral deficits in mice [17, 18, 44]. Cell type-specific conditional knockout of the translational repressor 4E-BP2 in GABAergic inhibitory neurons is sufficient to induce autistic-like phenotypes, although analysis of protein synthesis was not performed [19]. Here, we show that cell-type specific deletion of *Fmr1* in PV inhibitory neurons results in increased de novo protein synthesis in these cells in the mPFC. In addition, we found impaired social novelty behavior in *Fmr1*^−/y^-PV mice. PV neuron activity in mPFC has been implicated in mediating social behavior in mice [22, 45, 46]; therefore, it is not surprising that exaggerated protein synthesis in these cells may be associated with deficits in social behavior, as in our studies. On the other hand, de novo protein synthesis in hippocampal PV neurons was decreased rather than elevated upon cell type-specific deletion of *Fmr1,* which is inconsistent with the cellular function of FMRP as a translational repressor. In addition, this contrasted with elevated de novo protein synthesis observed in PV-positive neurons in *Fmr1*^−/y^ mouse hippocampus (Fig. 1D). These discrepancies could be driven by altered activity in another brain region in *Fmr1*^−/y^-PV mice, as global *Fmr1* deletion may have different circuit-level effects compared to cell type-specific *Fmr1* ablation. Furthermore, we observed elevated PV expression in *Fmr1*^−/y^-PV hippocampus but not in the *Fmr1*^−/y^ mouse hippocampus, which may also be driven by differences in the activity in PV-positive cells in these models as it known that parvalbumin expression can be regulated by activity [47, 48].

Signaling by group 1 mGluRs stimulates protein synthesis in response to synaptic activity and in *Fmr1*^−/y^ mice protein synthesis is exaggerated and not responsive to further induction by group 1 mGluR activation [49, 50]. Several receptor-mediated signal transduction pathways that regulate neuronal protein synthesis, including muscarinic acetylcholine receptors, dopamine D1/5 receptors and tyrosine kinase receptor B, were also found to be dysregulated in *Fmr1*^−/y^ mice [51–53]. Thus, it’s possible that in PV-positive neurons in *Fmr1*^−/y^-PV mice, mTORC1 signaling is decoupled from protein synthesis. In the hippocampus, we observed decreased protein synthesis despite elevated rpS6 phosphorylation which may indicate that mTORC1 activation was unable to elicit an increase in protein synthesis. On the other hand, in mPFC we observed elevated protein synthesis whereas rpS6 phosphorylation was unaltered, which may also suggest that protein synthesis is not responsive to mTORC1 signaling in PV-positive neurons in *Fmr1*^−/y^-PV mice.

In the context of global *Fmr1* deletion in *Fmr1*^−/y^ mice, several studies reported abnormal PV inhibitory neuron development and function. In *Fmr1*^−/y^ mouse somatosensory cortex, PV neurons showed altered expression and morphology, delayed maturation, and deficits in local excitation [54–57]. In addition, impaired visual discrimination in *Fmr1*^−/y^ mice, was shown to be correlated with decreased PV inhibitory neuron activity in the primary visual cortex and can be rescued by normalizing PV neuron activity [58]. Deficits in SOM neurons have not been described in *Fmr1*^−/y^ mice. Our data indicate that de novo protein synthesis was elevated in SOM and PV inhibitory neurons in the *Fmr1*^−/y^ mouse hippocampus. Furthermore, we found that cell type-specific deletion of *Fmr1* in PV, but not SOM, neurons was associated with dysregulated protein synthesis and can recapitulate some FXS-like behavioral deficits. How might dysregulated protein synthesis in PV inhibitory neurons lead to behavioral deficits? PV inhibitory neurons regulate pyramidal neuron activity and play an important role in gating cortical excitation/inhibition (E/I) balance, which is critical for proper cortical function as E/I imbalance is associated with multiple psychiatric conditions [59, 60]. FMRP has been shown to regulate the expression of Kv4.2 potassium channels and surface expression of Cav2.2 calcium channels [61, 62]. FMRP also interacts with Slack and BK potassium channels to modulate their activity [63, 64]. In addition, mRNAs of numerous ion channel subunits, including N-type, L-type, and R-type and T-type calcium channel, among others, have been identified as putative FMRP targets [9, 65]. Altered expression or function of these proteins in the absence of FMRP could potentially lead to altered intrinsic cell excitability [66]. Previous studies in *Fmr1*^−/y^ mice suggest neuronal and circuit hyperexcitability in several brain regions, which is in line with clinical features of sensory hypersensitivity observed in FXS individuals [66]. Furthermore, PV neurons appear to be especially sensitive to insults, including disruption of ASD-associated genes as deficits in PV neurons were observed in mouse models harboring deletion of other ASD-associated genes including *Cntnap2*, *Mecp2*, *Nlgn3* and *Shank3* and in human postmortem neocortical tissue from subjects with autism [67–70]. Thus, PV inhibitory neurons may contribute to mediating ASD pathophysiology and represent a potential therapeutic target in ASD and other psychiatric diseases.

We found that following threat conditioning, *Fmr1*^−/y^-PV mice spend significantly more time freezing in response to the auditory tone. In the basolateral amygdala, PV neuron activity during presentation of the CS was shown to correlate with enhanced learning [23]. Thus, our findings implicate FMRP in the molecular mechanisms underlying acquisition and retrieval of auditory threat memory and suggest that PV neuronal activity may be altered following *Fmr1* deletion as inhibition of PV neurons enhances US-induced freezing [23]. Because GABAergic inhibitory neuron activity is linked to anxiety, more studies are needed to uncover a potential role of FMRP in amygdala in anxiety-like behavior observed in FXS [71, 72].

## Limitations

The mouse models generated in this study using Cre-lox recombinase technology exhibit deletion of *Fmr1* in all parvalbumin or somatostatin-expressing cells, and although the majority of parvalbumin or somatostatin-expressing cells in cerebral cortex are thought to be GABAergic inhibitory neurons [73–75], some parvalbumin-expressing cells also are glutamatergic projection neurons, such as those in superior colliculus and hypothalamus [76, 77]. FMRP expression is highest in hippocampus and cortex, but it is also found in superior colliculus and hypothalamus [33]. Thus, additional studies in brain region-specific knockout of FMRP in parvalbumin-expressing neurons or rescue experiments in specific brain regions are needed to establish a causal relationship between cellular deficits and FXS-like behaviors.

## Conclusions

In conclusion, we found that FMRP was expressed in PV and SOM inhibitory neurons in mouse hippocampus and global de novo protein synthesis was elevated in these cells in *Fmr1*^−/y^ mice. Furthermore, we show that cell type-specific *Fmr1* deletion in PV-expressing neurons was associated with a discrete set of FXS-like behavioral deficits and perturbation of de novo protein synthesis in these cells. Ablating *Fmr1* in SOM-positive neurons did not result in behavioral deficits and did not impact global de novo protein synthesis in SOM-positive cells. Our findings add to the understanding of cell type-specific role of FMRP in two major inhibitory neuron populations in mediating FXS-like behavioral abnormalities.

## Supplementary Information

**Additional file 1. Supplemental Figures S1-S5. Figure S1.** FMRP localizes to parvalbumin (PV) and somatostatin (SOM)-expressing neurons in mouse hippocampus. **Figure S2.** FMRP expression and rotarod testing in Fmr1-/y-PV mice. **Figure S3.** Object recognition, MWM reversal, and visible MWM behavior of Fmr1-/y-PV mice. **Figure S4.** FMRP expression and behavoioral testing of Fmr1-/y-SOM mice. **Figure S5.** Phosphorylation of ribosomal protein S6 (rpS6) was elevated in PV-positive neurons in the hippocampus but not mPFC of WT and Fmr1-/y-PV mouse.

**Additional file 2. Table S1.** Summary of molecular changes in Fmr1-/y, Fmr1-/y-PV and Fmr1-/y-SOM mice.

## Data Availability

All data generated or analyzed during this study are included in this published article (and its supplementary information files).

## Abbreviations

AHA: Azidohomoalanine
ASD: Autism spectrum disorder
cKO: Conditional KO
CS: Conditioned stimulus
DSM-V: Diagnostic and statistical manual of mental disorders
EPM: Elevated plus maze
FMRP: Fragile X mental retardation protein
FUNCAT: Fluorescent non-canonical amino acid tagging
FXS: Fragile X syndrome
GABA: γ-Aminobutyric acid
ID: Intellectual disability
KO: Knockout
LTM: Long-term memory
mPFC: Medial prefrontal cortex
mTORC1: Mammalian target of rapamycin complex 1
MWM: Morris water maze
OLM: Object location memory
PFA: Paraformaldehyde
PI: Preference index
PV: Parvalbumin
rpS6: Ribosomal protein S6
SOM: Somatostatin
STM: Short-term memory
US: Unconditioned stimulus
WT: Wild type
3CSI: 3-Chamber social interaction
4E-BP2: Eukaryotic translation initiation factor 4E-binding protein 2
