# Supplementary material for: Deletion of Fmr1 in parvalbumin-expressing neurons results in dysregulated translation and selective behavioral deficits associated with fragile X syndrome
Source: Mol Autism. 2022 Jun 29;13:29. doi: 10.1186/s13229-022-00509-2 (PMC9245312; doi:10.1186/s13229-022-00509-2)
Supplement: Supplementary file 1 — Additional file 1. Supplemental Figures S1-S5. Figure S1. FMRP localizes to parvalbumin (PV) and somatostatin (SOM)-expressing neurons in mouse hippocampus. Figure S2. FMRP expression and rotarod testing in Fmr1-/y-PV mice. Figure S3. Object recognition, MWM reversal, and visible MWM behavior of Fmr1-/y-PV mice. Figure S4. FMRP expression and behavoioral testing of Fmr1-/y-SOM mice. Figure S5. Phosphorylation of ribosomal protein S6 (rpS6) was elevated in PV-positive neurons in the hippocampus but not mPFC of WT and Fmr1-/y-PV mouse. [file 13229_2022_509_MOESM1_ESM.pdf]

Figure S1

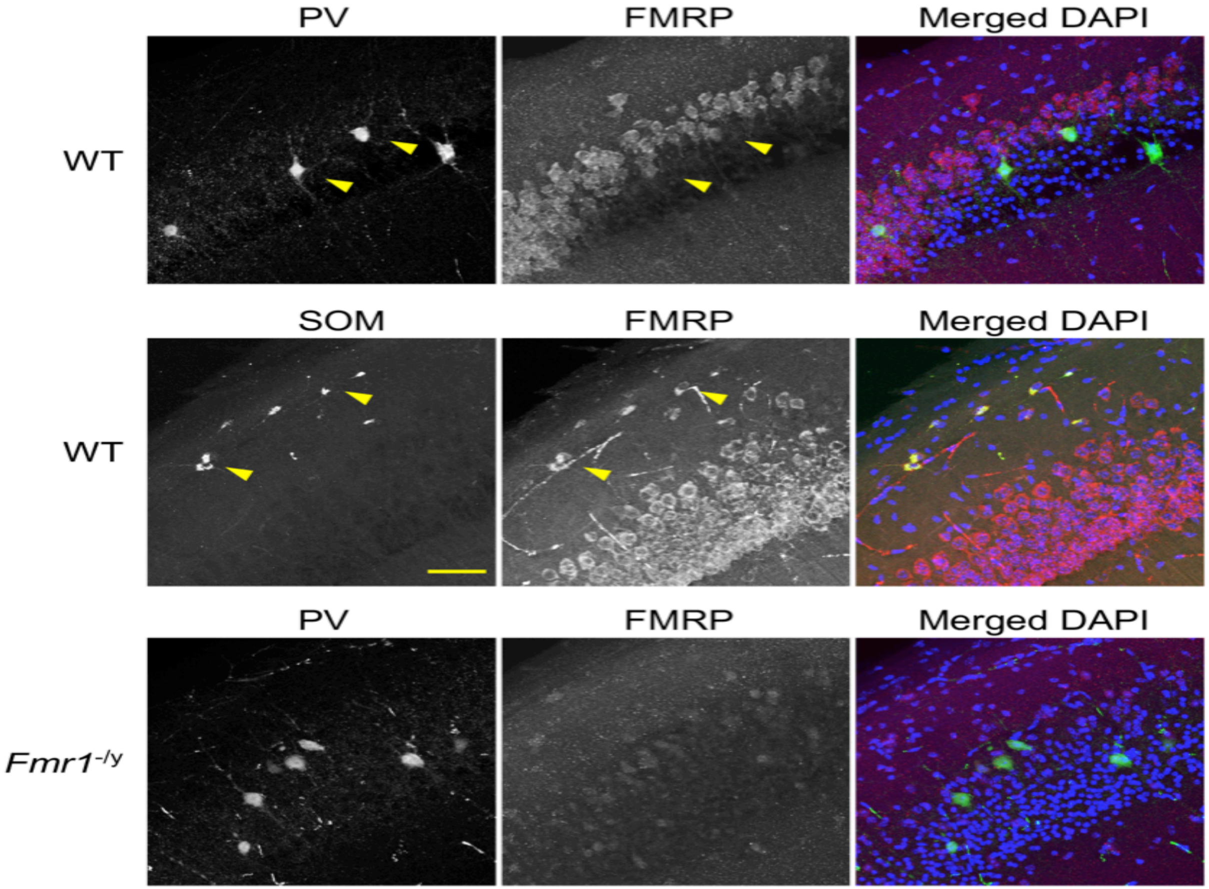

Figure S1. FMRP localizes to parvalbumin (PV) and somatostatin (SOM)-expressing neurons in mouse hippocampus. Representative images showing immunostaining of FMRP and PV (top) or SOM (middle) in WT mouse hippocampus. Arrows point to individual cells co-expressing FMRP and PV or SOM. Bottom panel shows absence of FMRP labeling in *Fmr1*$^{-/y}$ mouse hippocampus; Scale bar: 50 μm.

Figure S2

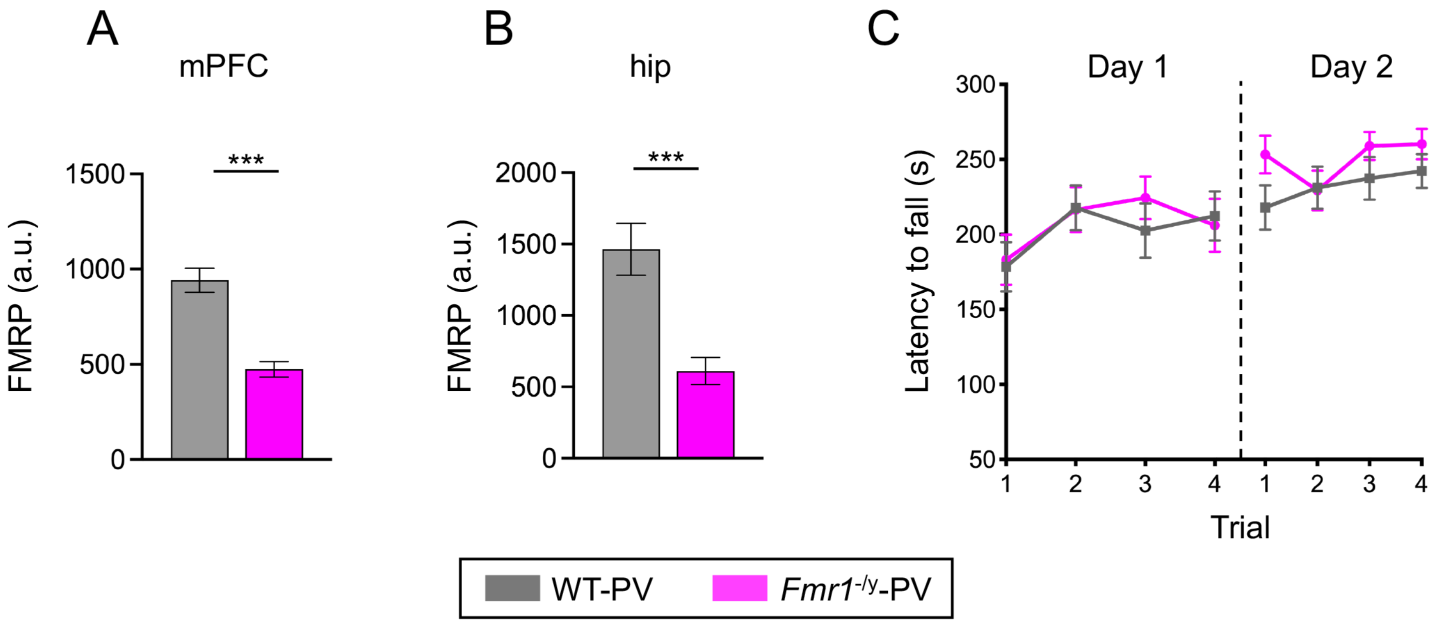

Figure S2. FMRP expression and rotarod testing in *Fmr1$^{-/y}$*-PV mice. Quantification of FMRP fluorescence in PV-expressing cells in WT-PV and *Fmr1$^{-/y}$*-PV mouse (A) mPFC and (B) hippocampus. (C) Rotarod test. *Fmr1$^{-/y}$*-PV and WT-PV mice showed no significant differences in motor coordination and learning in the rotarod test. Values represent mean ± SEM (n = 12-14 animals per genotype); RM two-way ANOVA followed by Bonferroni's multiple comparisons test.

Figure S3

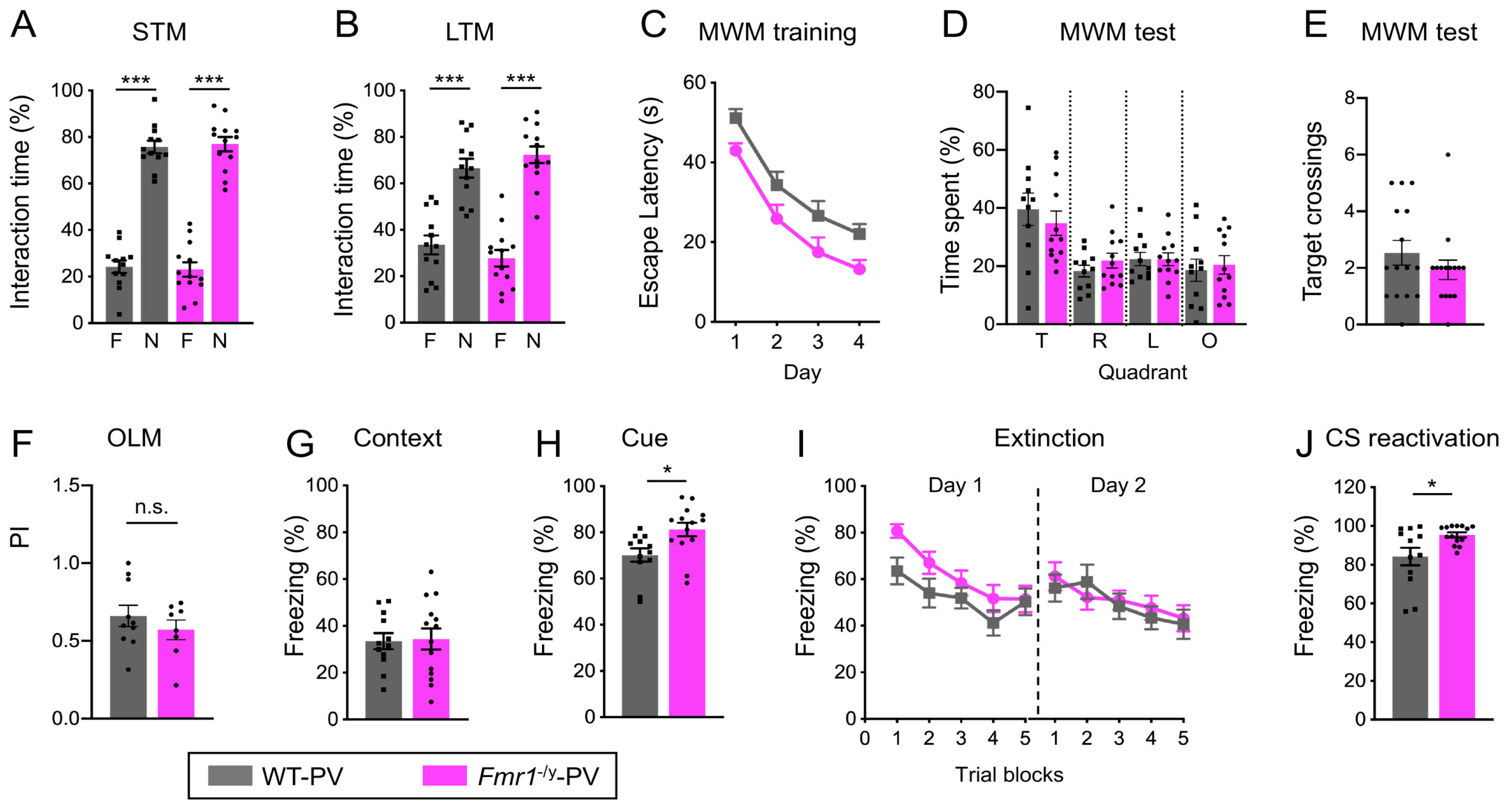

Figure S3. Object recognition, MWM reversal, and visible MWM behavior of *Fmr1*$^{-/y}$-PV mice. (A) Preference Index (PI) for a novel object during STM and LTM portions of object recognition test did not differ significantly between *Fmr1*$^{-/y}$-PV and WT-PV mice. Values represent mean ± SEM (n = 12-13 animals per genotype). (B) Escape latency during the reversal or flag test portions of MWM was not significantly different between *Fmr1*$^{-/y}$-PV and WT-PV mice. Values represent mean ± SEM (n = 15 animals per genotype); RM Two-way ANOVA followed by Bonferroni's multiple comparisons test, Student's *t* test.

Figure S4

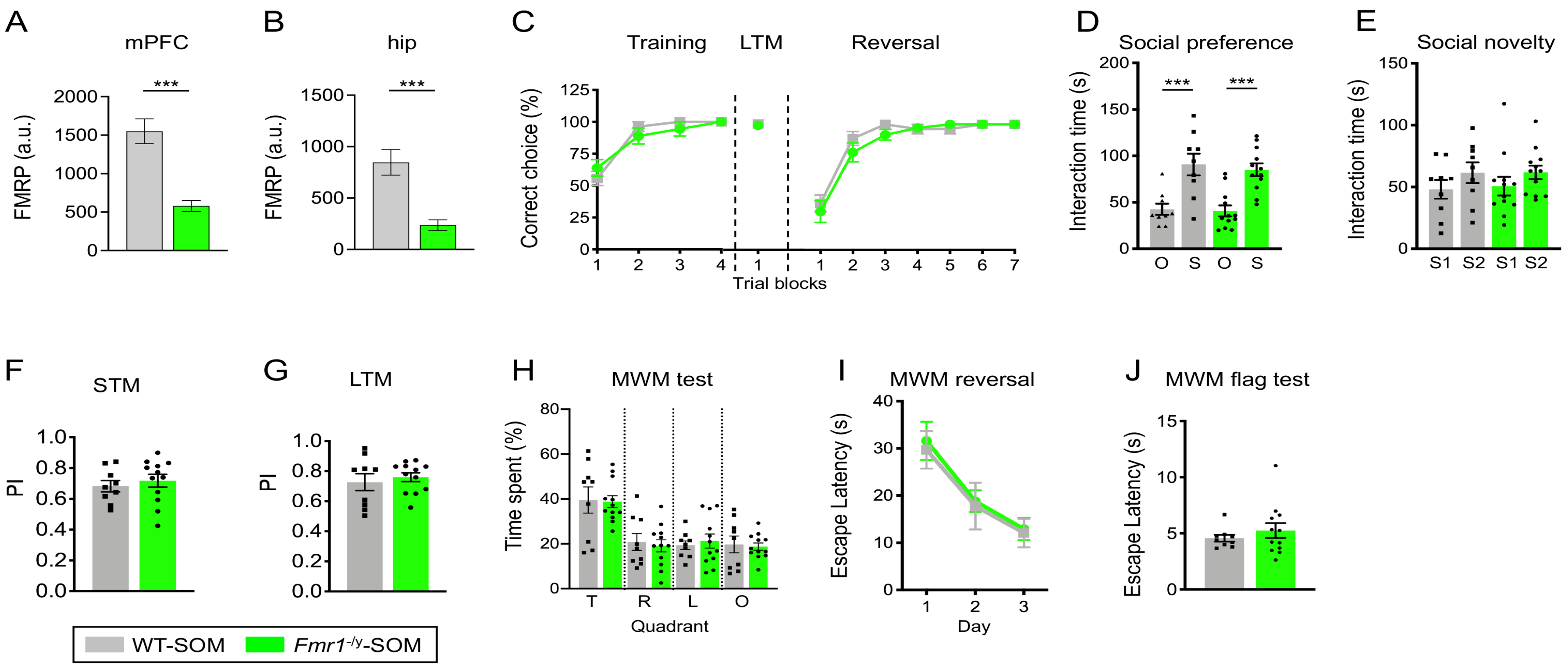

Figure S4. FMRP expression and behavoioral testing of *Fmr1*$^{-/y}$-SOM mice. Quantification of FMRP fluorescence in SOM-expressing cells in WT-SOM and *Fmr1*$^{-/y}$-SOM (A) mPFC and (B) hippocampus. Water-based Y maze, three chamber social preference and social novelty test, object recognition, MWM probe test, and visible MWM test were performed on *Fmr1*$^{-/y}$-SOM mice. (C) There was no significant difference in percentage of correct choices during the training, LTM, or reversal portions of the Y Maze task between WT-SOM and *Fmr1*$^{-/y}$-SOM mice. Values represent mean ± SEM (n = 9-12 animals per genotype. (D-E) WT-SOM and *Fmr1*$^{-/y}$-SOM mice spent more time exploring a stranger mouse (S) compared to an object (O) during social preference portion of the 3CSI Test (D). Both genotypes did not exhibit a preference for social novelty as the time spent exploring a novel stranger mouse (S2) compared to a familiar stranger mouse (S1) did not differ significantly (E). Values represent mean ± SEM (n = 9-12 animals per genotype). (F-G) PI for a novel object during STM (F) or LTM (G) portions of object recognition test was not significantly different between the genotypes. Values represent mean ± SEM (n = 9-12 animals per genotype). (H-J) Target quadrant occupancy (H), frequency of platform crossings during 60 s probe trial (I) or during flag test (J) were not significantly different between *Fmr1*$^{-/y}$-SOM and WT-SOM mice. Values represent mean ± SEM (n = 9-12 animals per genotype); ***$p < 0.001$, two-way ANOVA or RM two-way ANOVA followed by Bonferroni's multiple comparisons test, Student's *t* test.

Figure S5

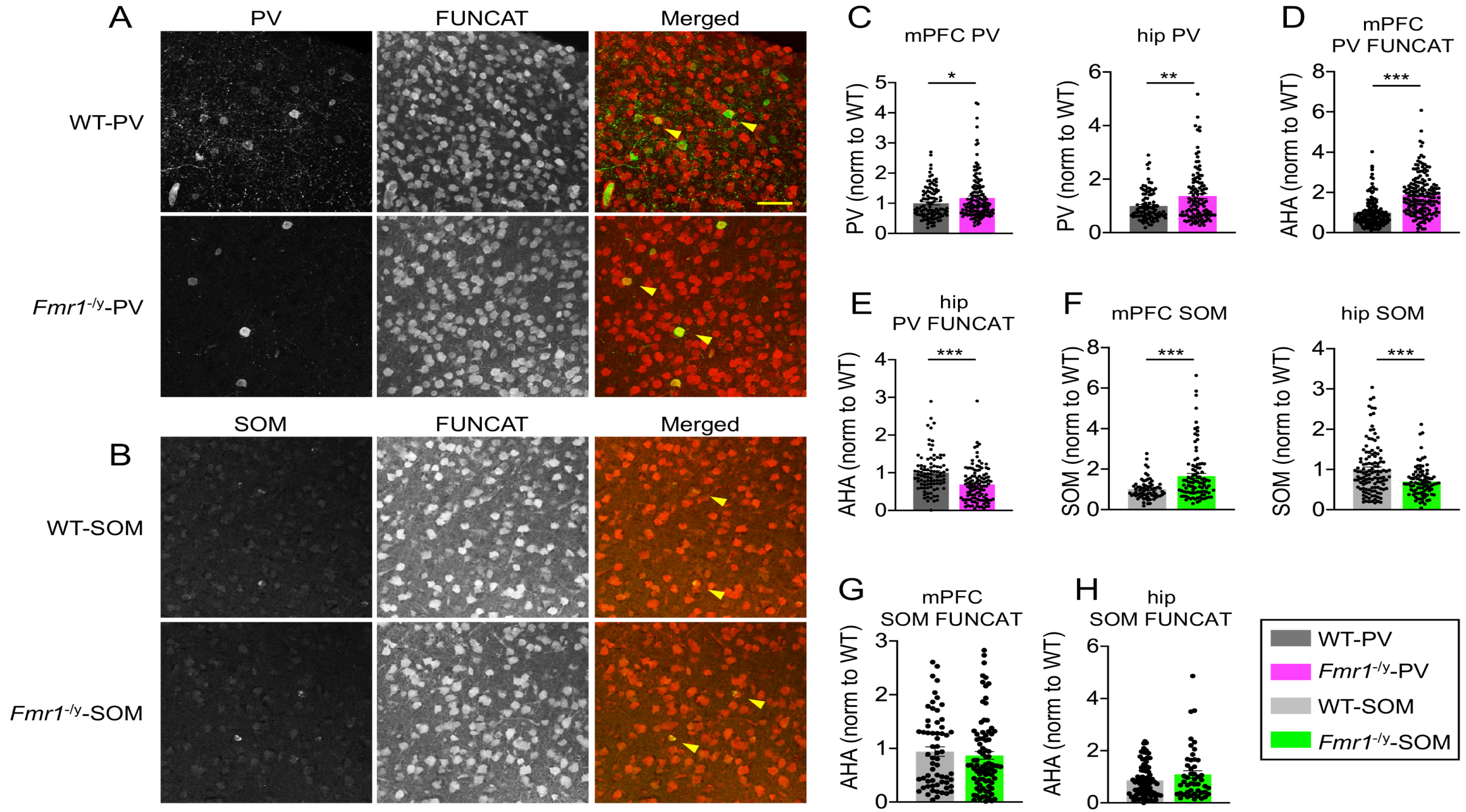

Figure S5. Phosphorylation of ribosomal protein S6 (rpS6) was elevated in PV-positive neurons in the hippocampus but not mPFC of WT and *Fmr1*$^{-/y}$-PV mouse. (A) Representative images of PV, rpS6 phosphorylated at serine 235 and 236 (p-S6 235/6) and neuronal marker NeuN staining from WT-PV and *Fmr1*$^{-/y}$-PV mouse hippocampus. (B-C) Quantification of p-S6 235/6 in PV-positive neurons in the hippocampus (B) and mPFC (C) of WT-PV and *Fmr1*$^{-/y}$-PV mouse. Values represent mean ± SEM (hippocampus, n = 20-23 z-stacks, 9 sections, from 3 animals per genotype; mPFC, n = 20-27 z-stacks, 9 sections, from 3 animals per genotype); *p < 0.05 (Student's *t* test). Scale bar: 100 μm.
