# Supplementary material for: Deletion of Fmr1 in parvalbumin-expressing neurons results in dysregulated translation and selective behavioral deficits associated with fragile X syndrome
Source: Mol Autism. 2022 Jun 29;13:29. doi: 10.1186/s13229-022-00509-2 (PMC9245312; doi:10.1186/s13229-022-00509-2)
Supplement: Supplementary file 2 — Additional file 2. Table S1. Summary of molecular changes in Fmr1-/y, Fmr1-/y-PV and Fmr1-/y-SOM mice. [file 13229_2022_509_MOESM2_ESM.docx]

**Table S1** Summary of molecular changes in *Fmr1*^−/y^*, Fmr1*^−/y^-PV and *Fmr1*^−/y^-SOM mice

|  | ***Fmr1*^−/y^** | ***Fmr1*^−/y^-PV** | ***Fmr1*^−/y^-SOM** |
| --- | --- | --- | --- |
| PV expression  Hippocampus  mPFC | No change | Increased  Increased |  |
| SOM expression  Hippocampus  mPFC | No change |  | Decreased  Increased |
| PV- FUNCAT  Hippocampus  mPFC | Increased | Decreased  Increased |  |
| SOM-FUNCAT  Hippocampus  mPFC | Increased |  | No change  No change |
| Phospho-S6 (235/6)  Hippocampus  mPFC |  | Increased  No change |  |
